# Supplementary material for: Exploration of Compounds with 2-Phenylbenzo[d]oxazole Scaffold as Potential Skin-Lightening Agents through Inhibition of Melanin Biosynthesis and Tyrosinase Activity
Source: Molecules. 2024 Sep 2;29(17):4162. doi: 10.3390/molecules29174162 (PMC11396935; doi:10.3390/molecules29174162)
Supplement: Supplementary file 1 [file molecules-29-04162-s001.zip › molecules-3170295-supplementary.pdf]

Supporting Information

For

Exploration of compounds with a 2-phenylbenzo[*d*]oxazole scaffold as potential skin-lightening agents through inhibition of melanin biosynthesis and tyrosinase activity

Hee Jin Jung [a,1], Hyeon Seo Park [a,1], Hye Soo Park [a], Hye Jin Kim [a], Dahye Yoon [a], Yujin Park [b], Pusoon Chun [c], Hae Young Chung [d], Hyung Ryong Moon [a,*]

[a]*Department of Manufacturing Pharmacy, College of Pharmacy and Research Institute for Drug Development, Pusan National University, Busan 46241, Republic of Korea*

[b]*Department of Medicinal Chemistry, New Drug Development Center, Daegu-Gyeongbuk Medical Innovation Foundation, Daegu 41061, South Korea*

[c]*College of Pharmacy and Inje Institute of Pharmaceutical Sciences and Research, Inje University, Gimhae 50834, Gyeongnam, Republic of Korea*

[d]*Department of Pharmacy, College of Pharmacy and Research Institute for Drug Development, Pusan National University, Busan 46241, Republic of Korea*

Contents

S1. ^{1}H NMR spectrum of analog **1** 4

S2. ^{13}C NMR spectrum of analog **1** 5

S3. ^{1}H NMR spectrum of analog **2** 6

S4. ^{13}C NMR spectrum of analog **2** 7

S5. ^{1}H NMR spectrum of analog **3** 8

S6. ^{13}C NMR spectrum of analog **3** 9

S7. ^{1}H NMR spectrum of analog **4** 10

S8. ^{13}C NMR spectrum of analog **4** 11

S9. ^{1}H NMR spectrum of analog **5** 12

S10. ^{13}C NMR spectrum of analog **5** 13

S11. ^{1}H NMR spectrum of analog **6** 14

S12. ^{13}C NMR spectrum of analog **6** 15

S13. ^{1}H NMR spectrum of analog **7** 16

S14. ^{13}C NMR spectrum of analog **7** 17

S15. ^{1}H NMR spectrum of analog **8** 18

S16. ^{13}C NMR spectrum of analog **8** 19

S17. ^{1}H NMR spectrum of analog **9** 20

S18. ^{13}C NMR spectrum of analog **9** 21

S19. ^{1}H NMR spectrum of analog **10** 22

S20. ^{13}C NMR spectrum of analog **10** 23

S21. ^{1}H NMR spectrum of analog **11**.. 24

S22. ^{13}C NMR spectrum of analog **11** 25

S23. ^{1}H NMR spectrum of analog **12** 26

S24. ^{13}C NMR spectrum of analog **12** 27

S25. ^{1}H NMR spectrum of analog **13** 28

S26. ^{13}C NMR spectrum of analog **13** 29

S27. ^{1}H NMR spectrum of analog **14** 30

S28. ^{13}C NMR spectrum of analog **14** 31

S29. ^{1}H NMR spectrum of analog **15** 32

S30. ^{13}C NMR spectrum of analog **15** 33

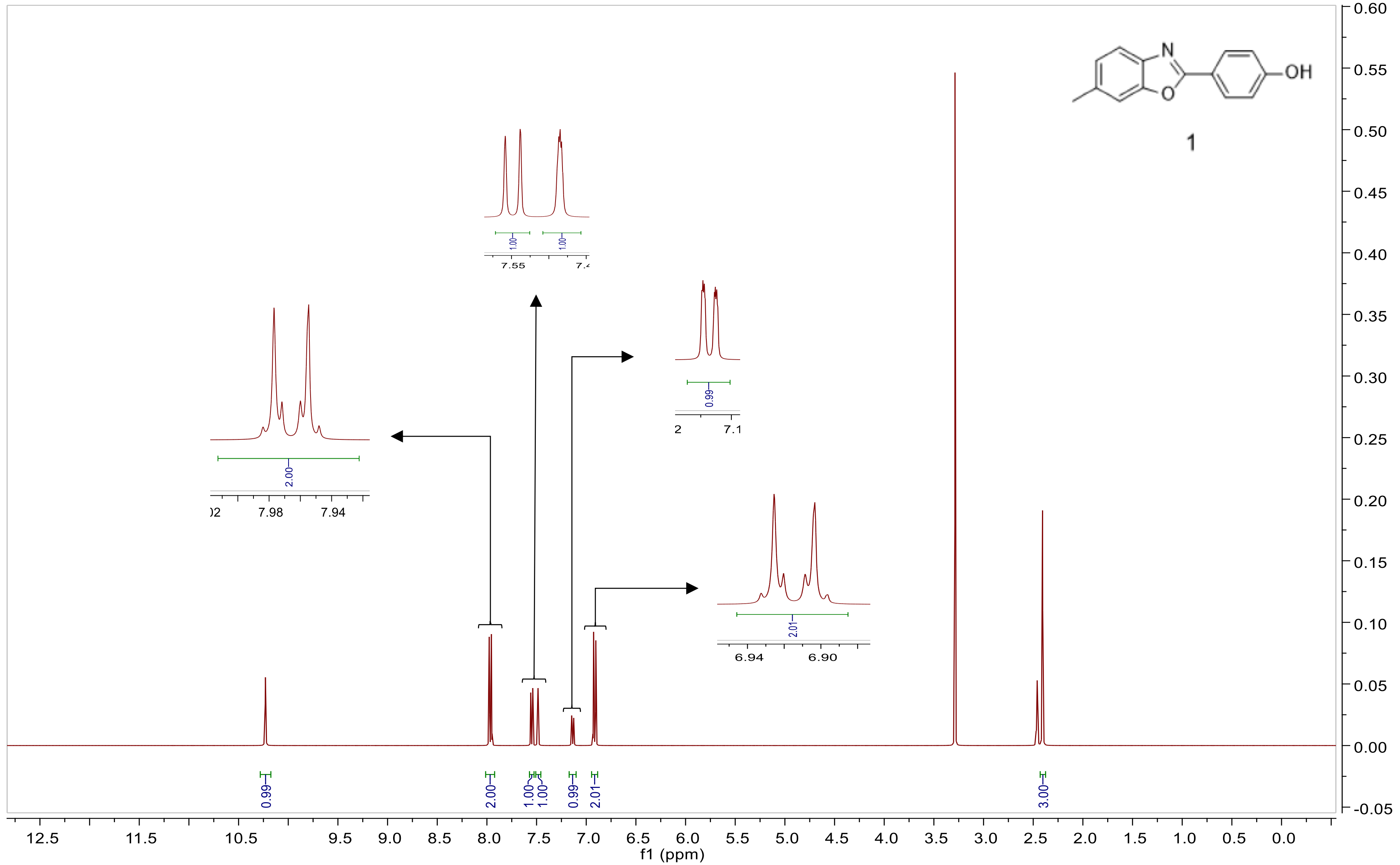

S1. ^{1}H NMR spectrum of analog **1**

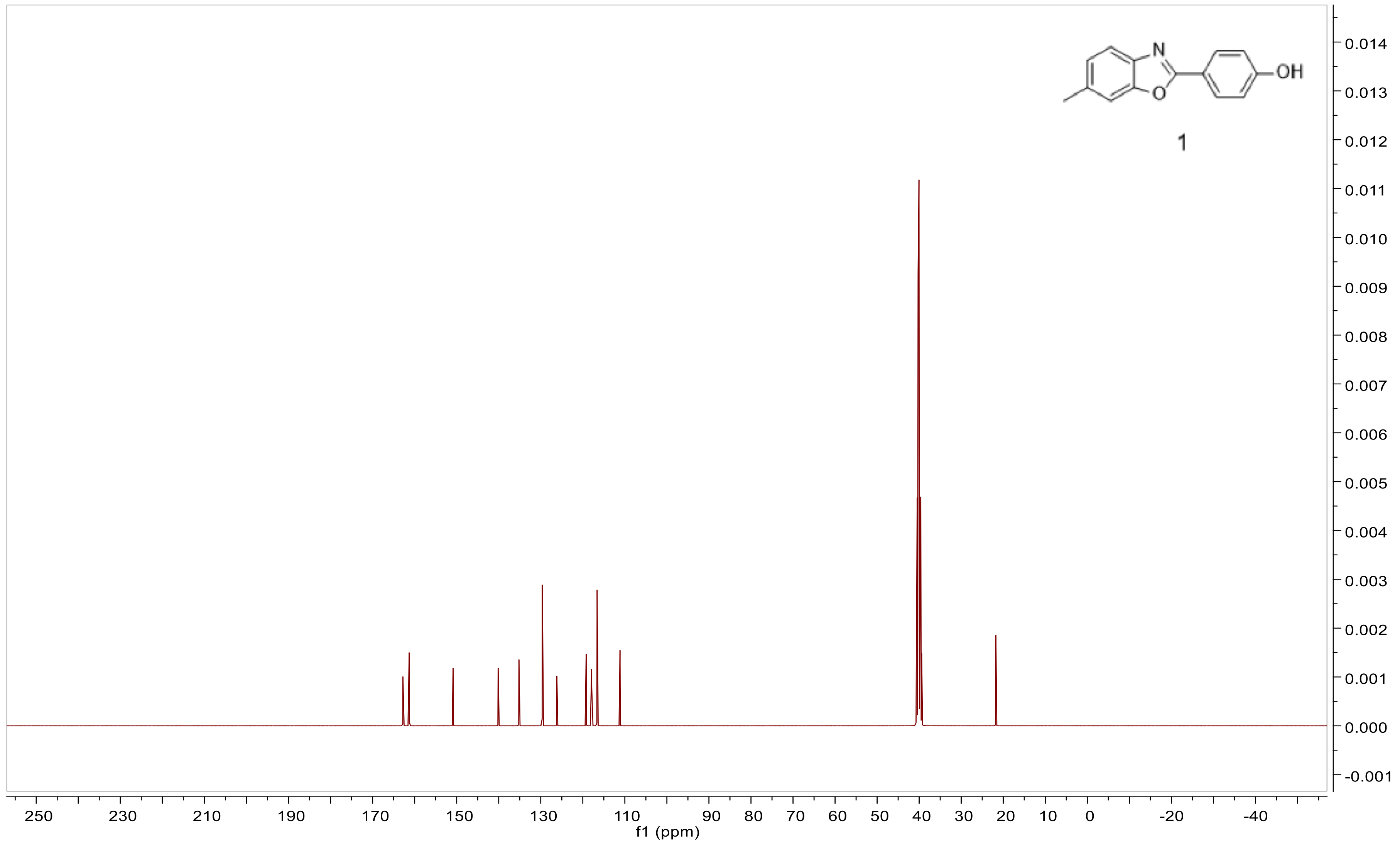

S2. ^{13}C NMR spectrum of analog **1**

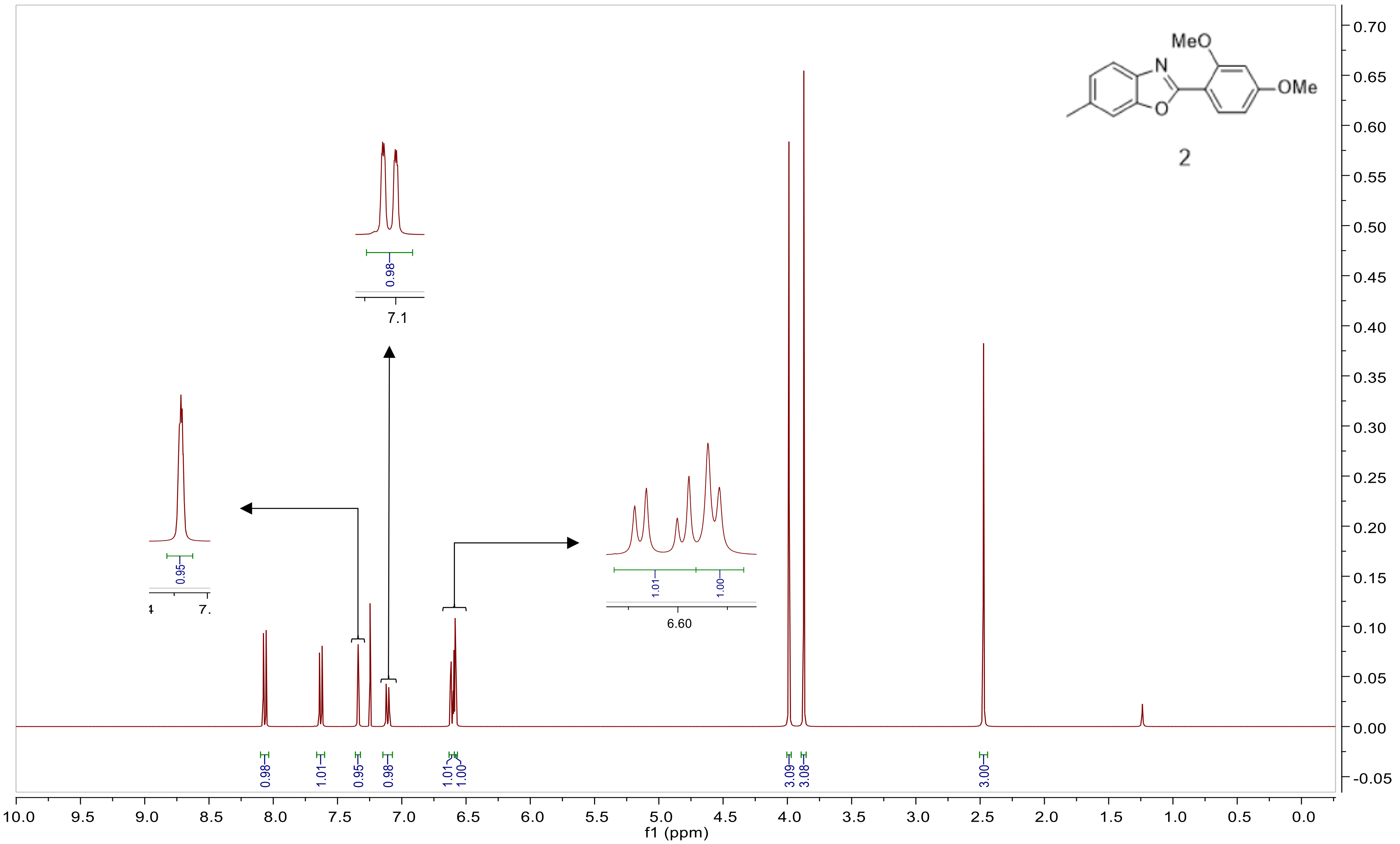

S3. ^{1}H NMR spectrum of analog **2**

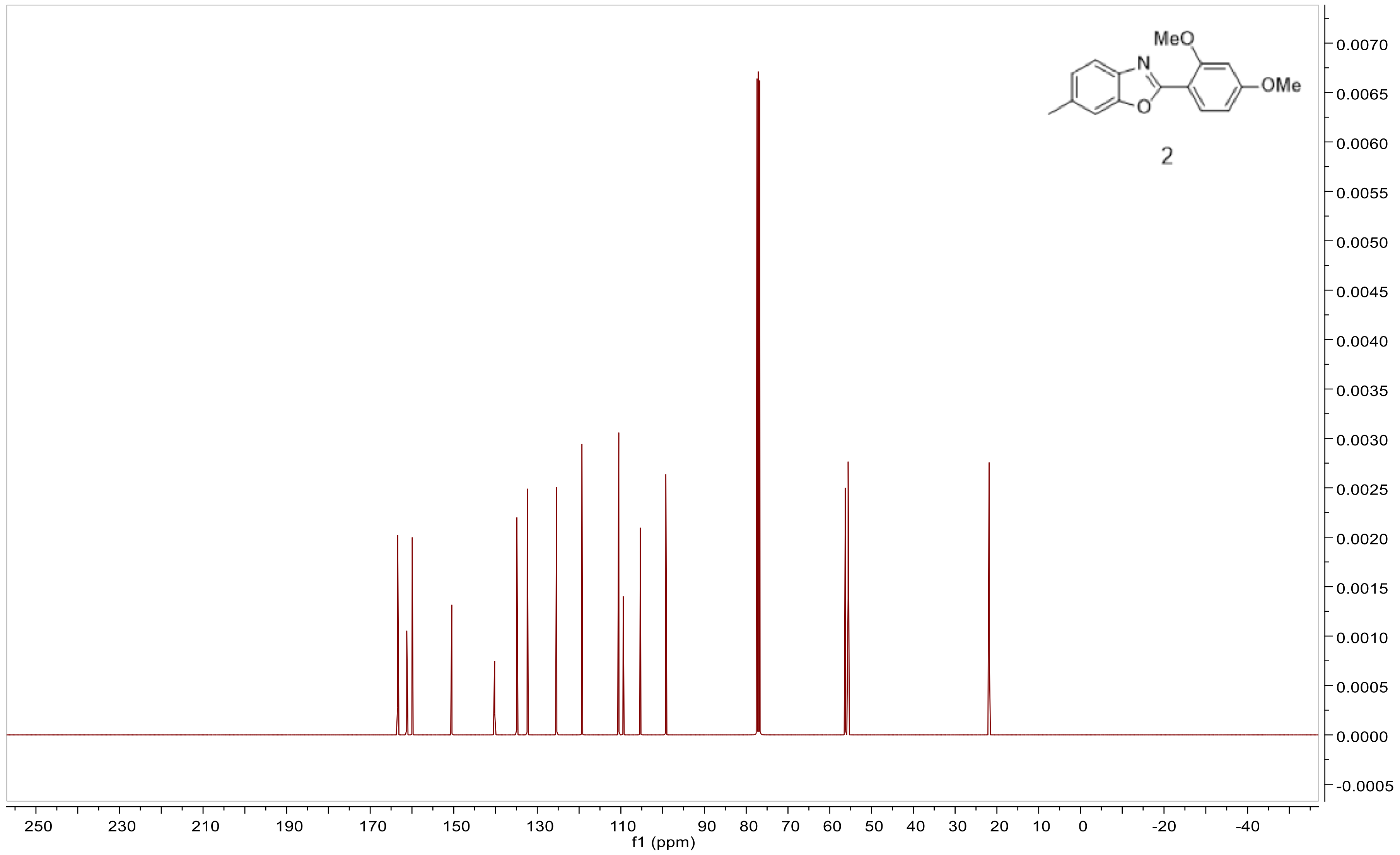

S4. ^{13}C NMR spectrum of analog **2**

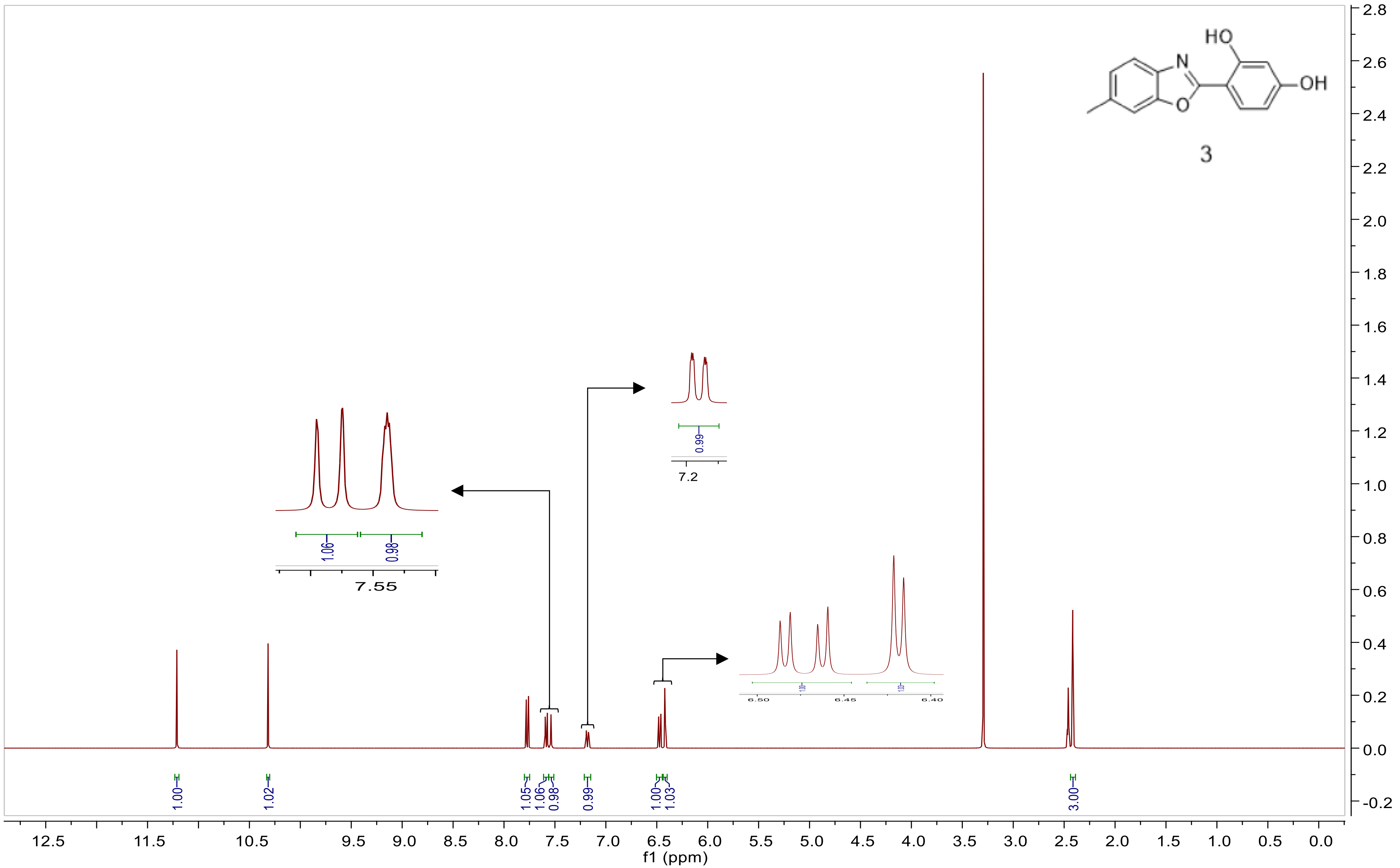

S5. ^{1}H NMR spectrum of analog **3**

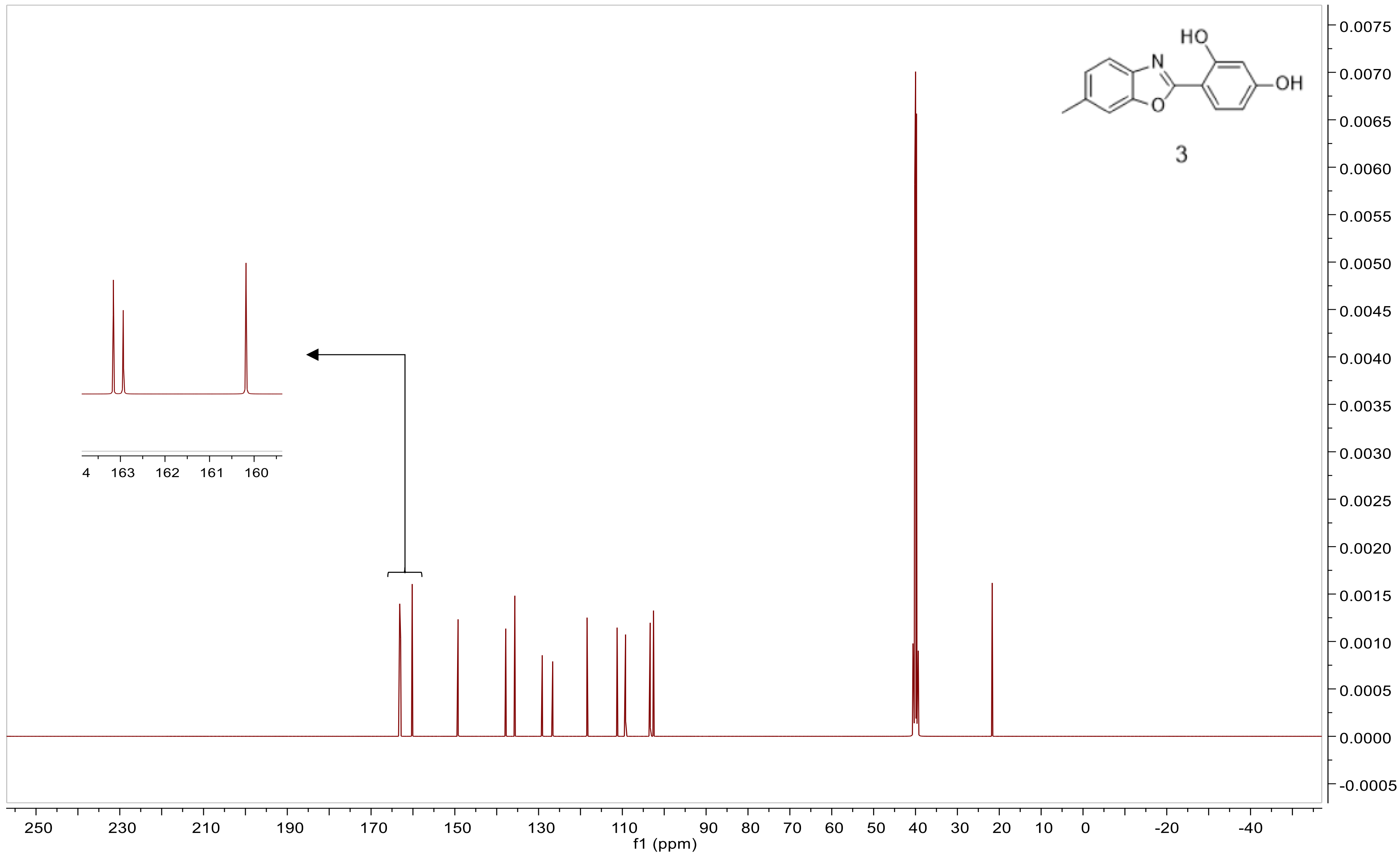

S6. ^{13}C NMR spectrum of analog **3**

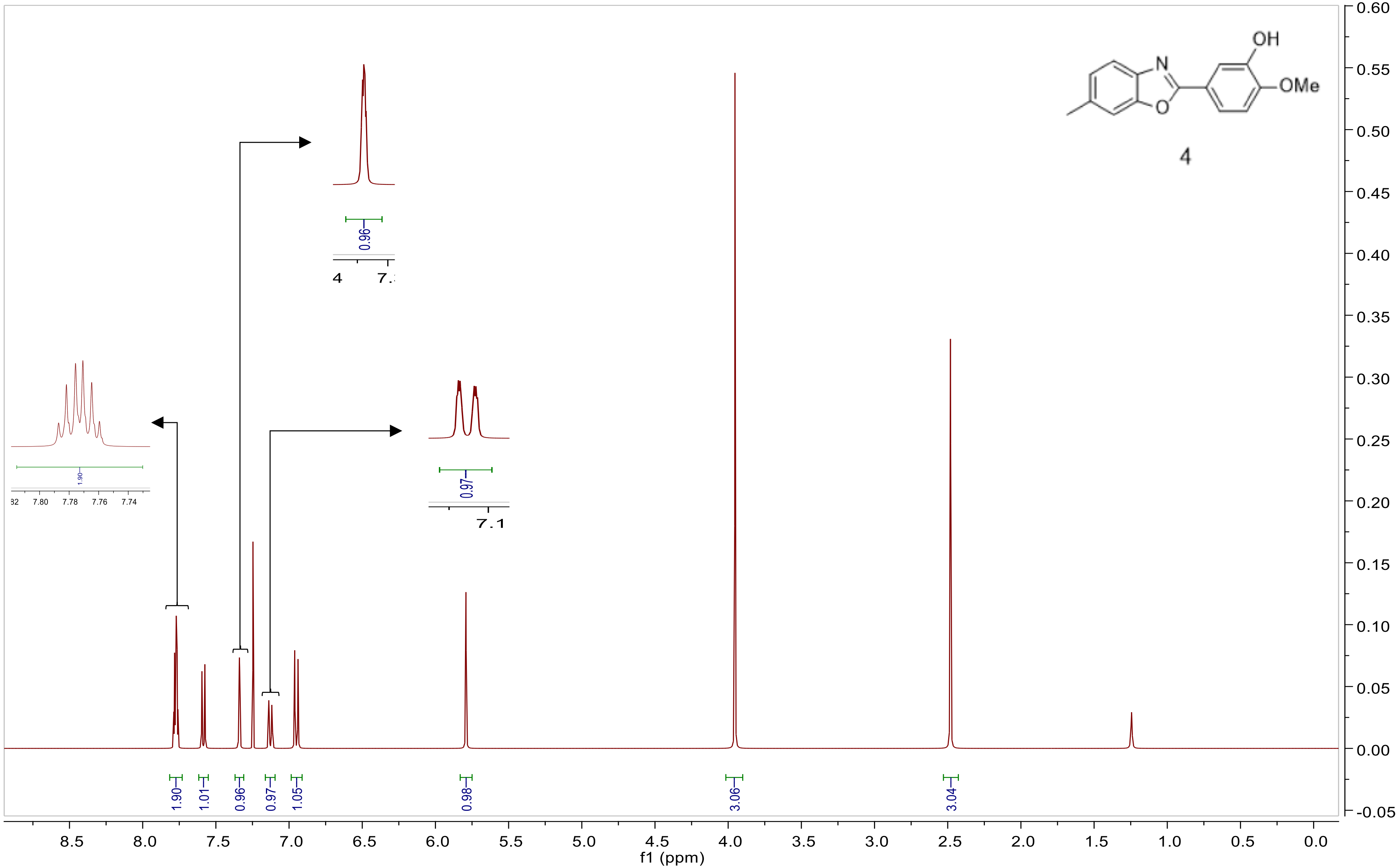

S7. ^{1}H NMR spectrum of analog **4**

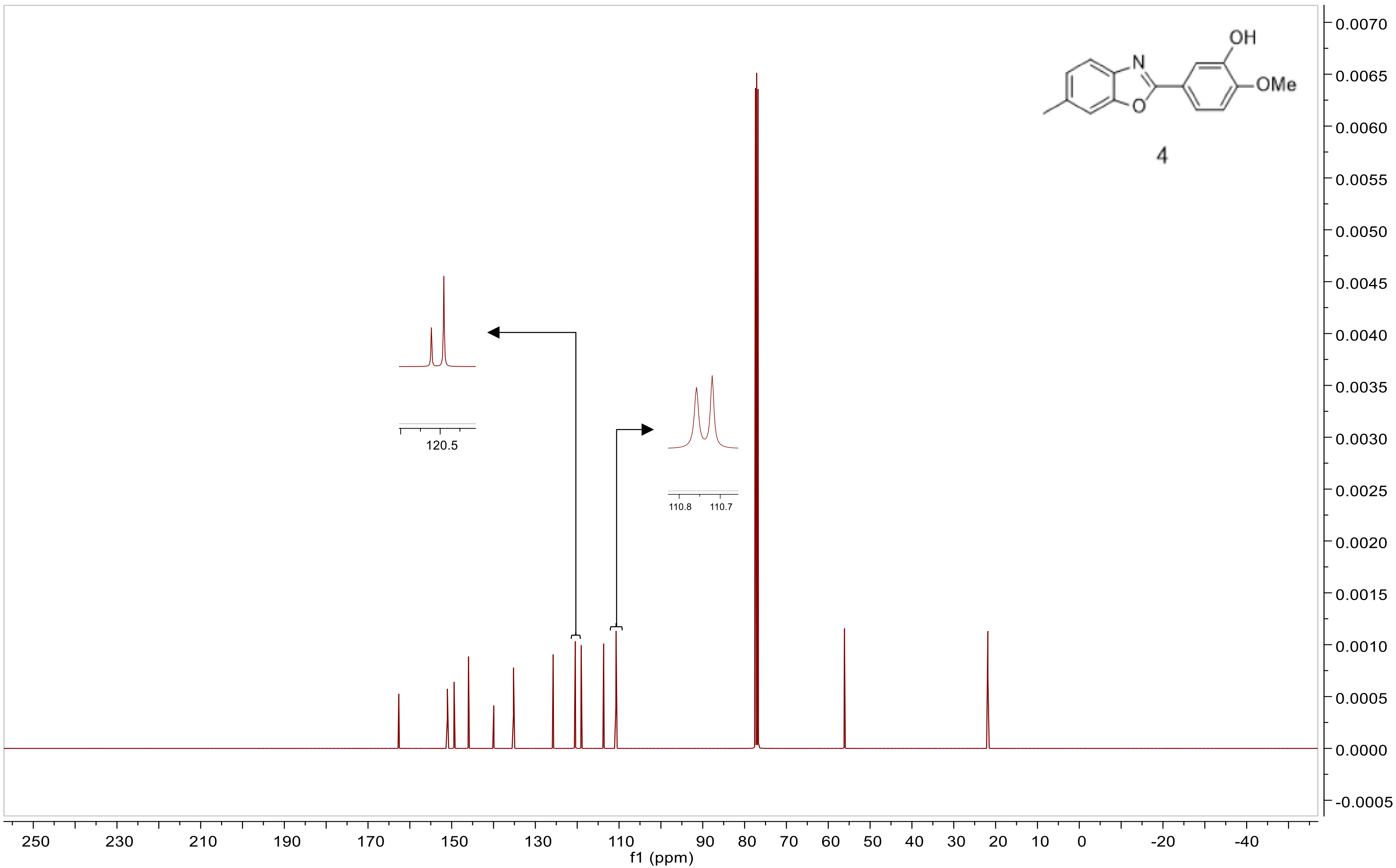

S8. ^{13}C NMR spectrum of analog **4**

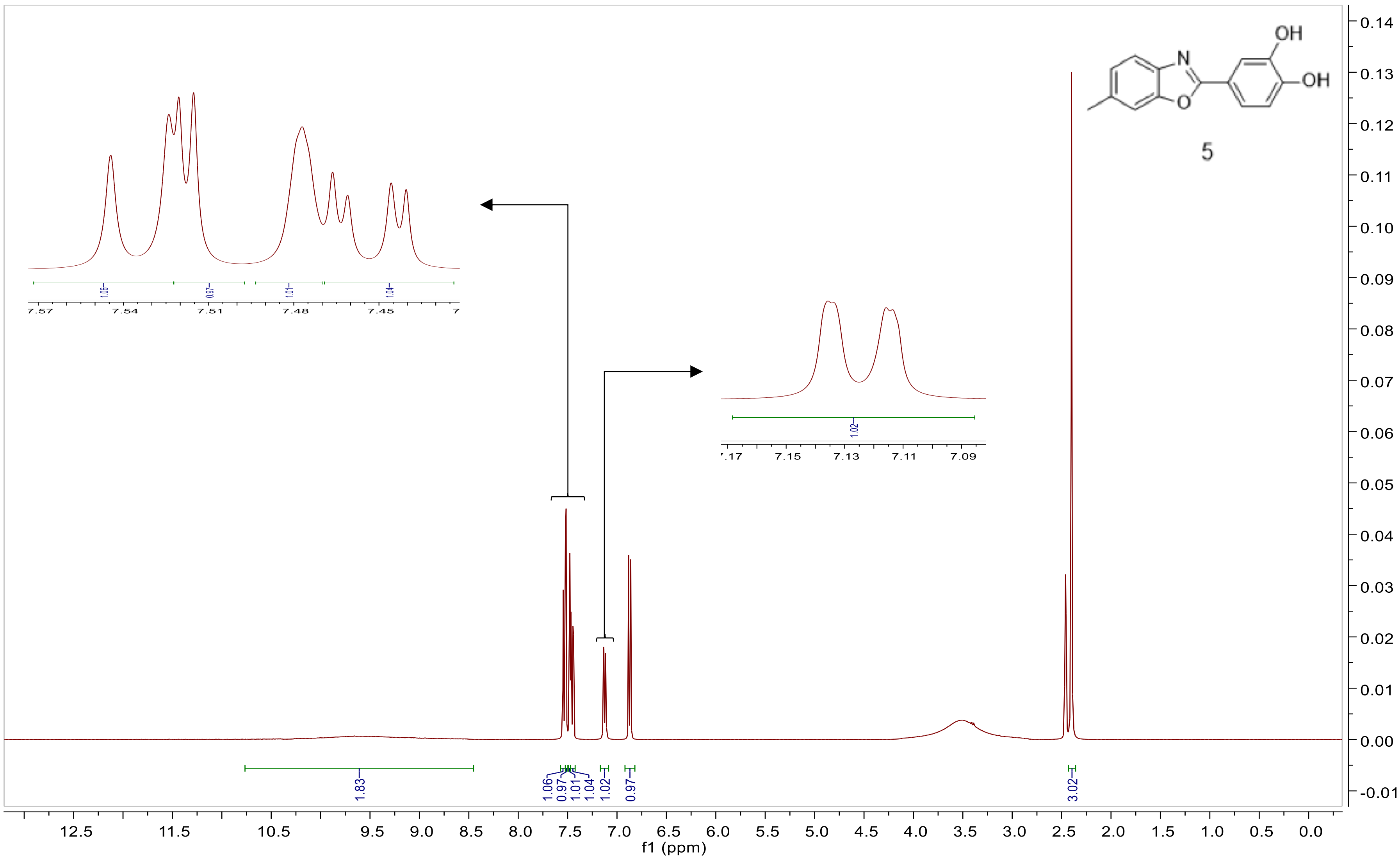

S9. ^{1}H NMR spectrum of analog **5**

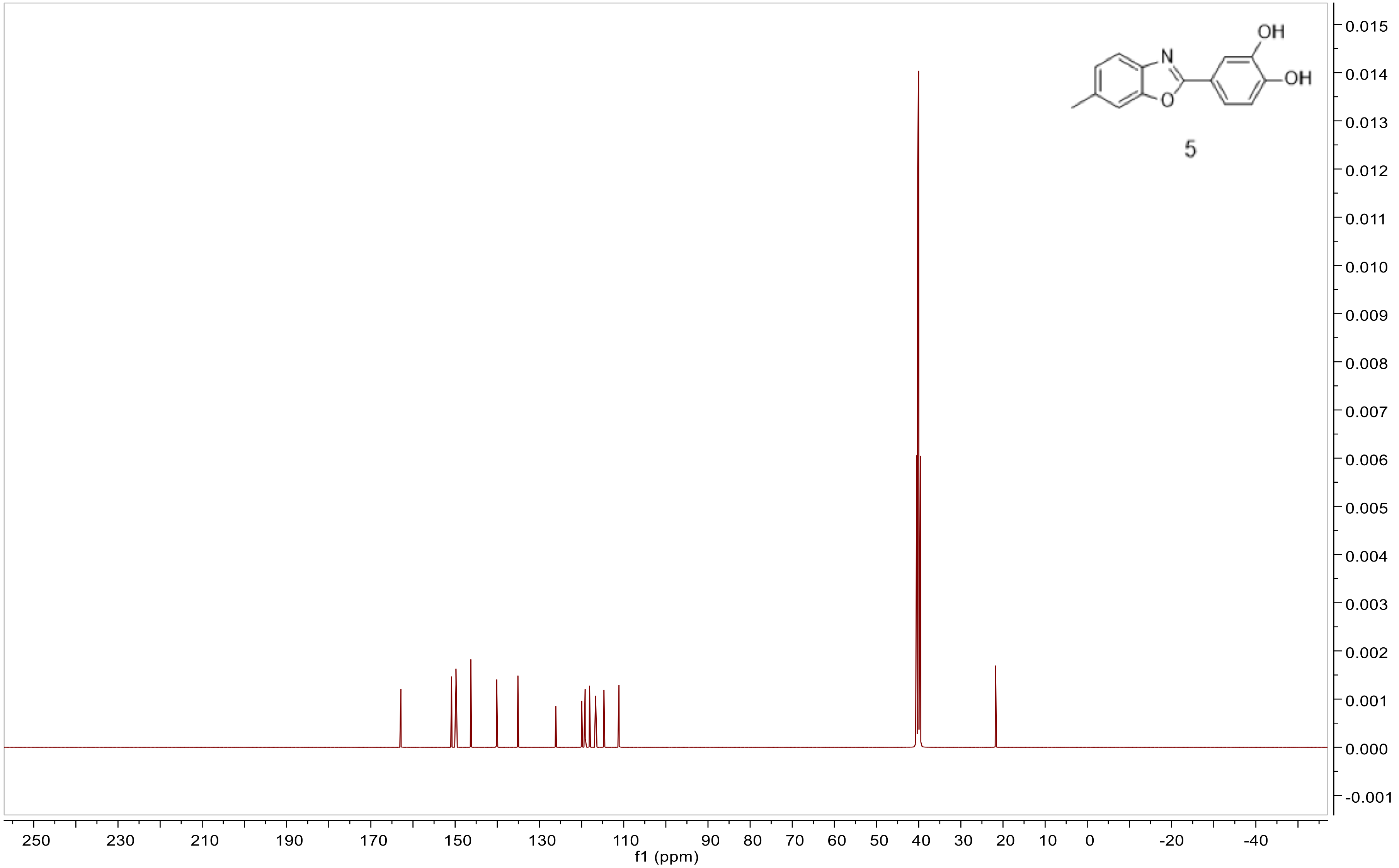

S10. ^{13}C NMR spectrum of analog **5**

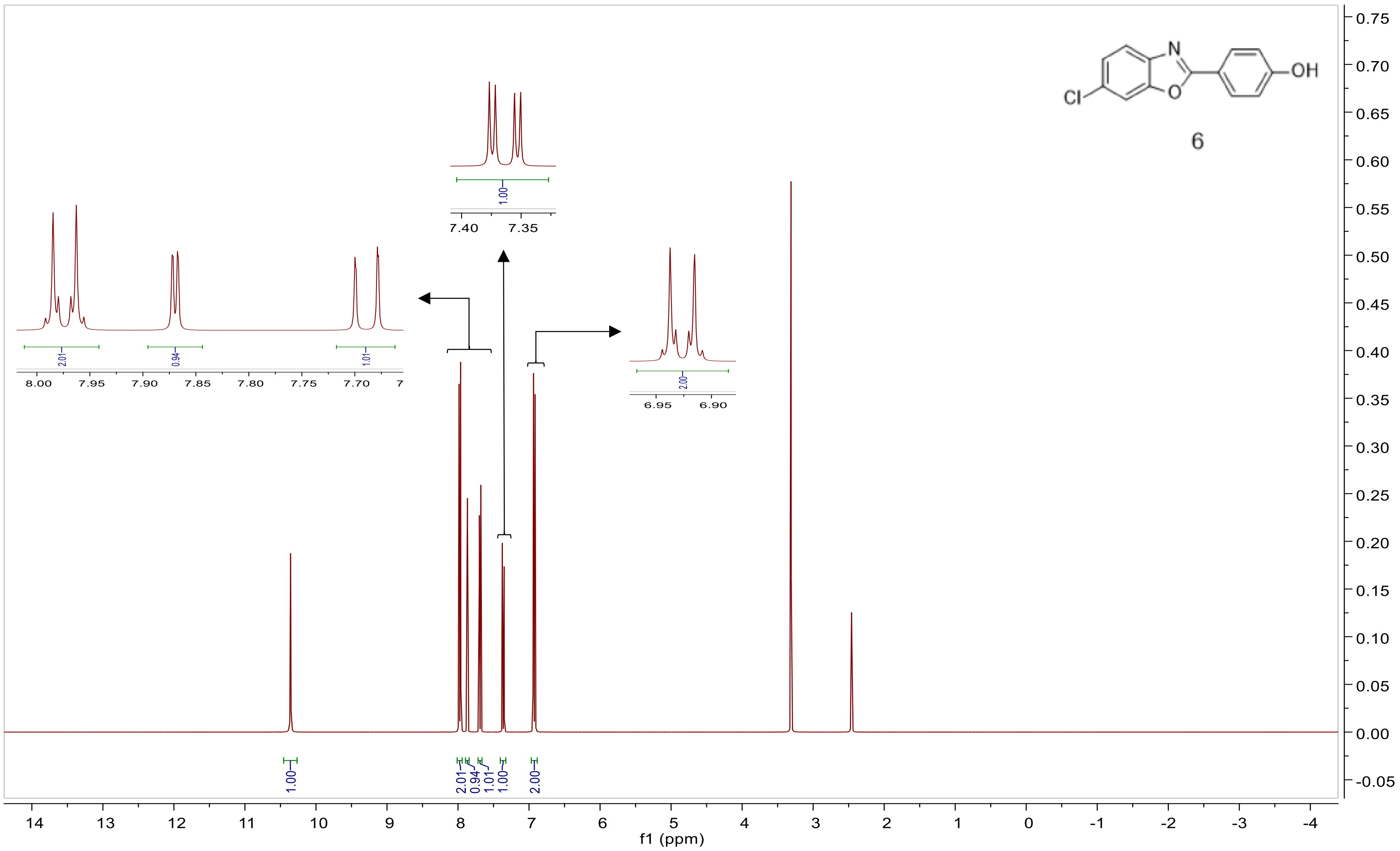

S11. ^{1}H NMR spectrum of analog **6**

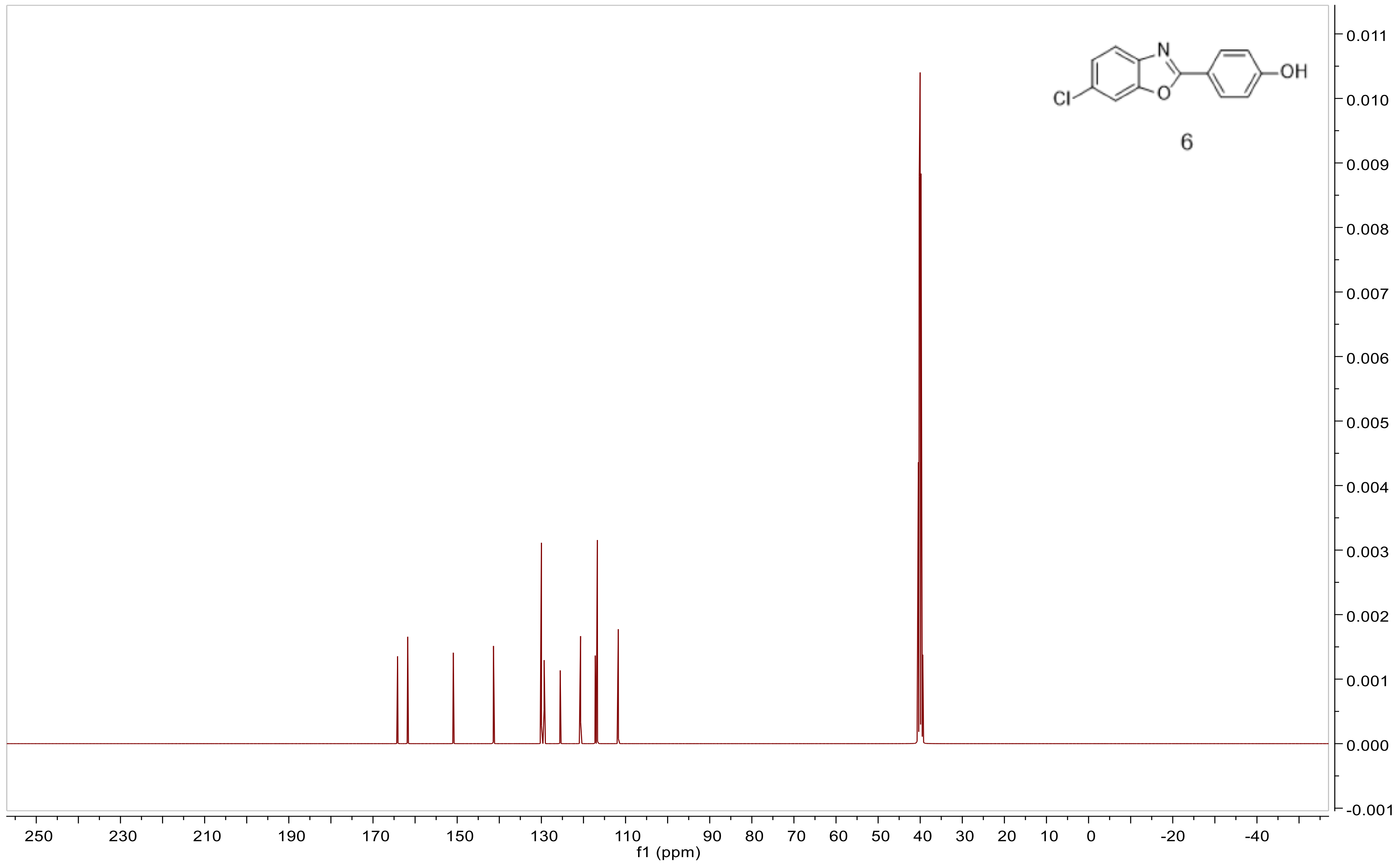

S12. ^{13}C NMR spectrum of analog **6**

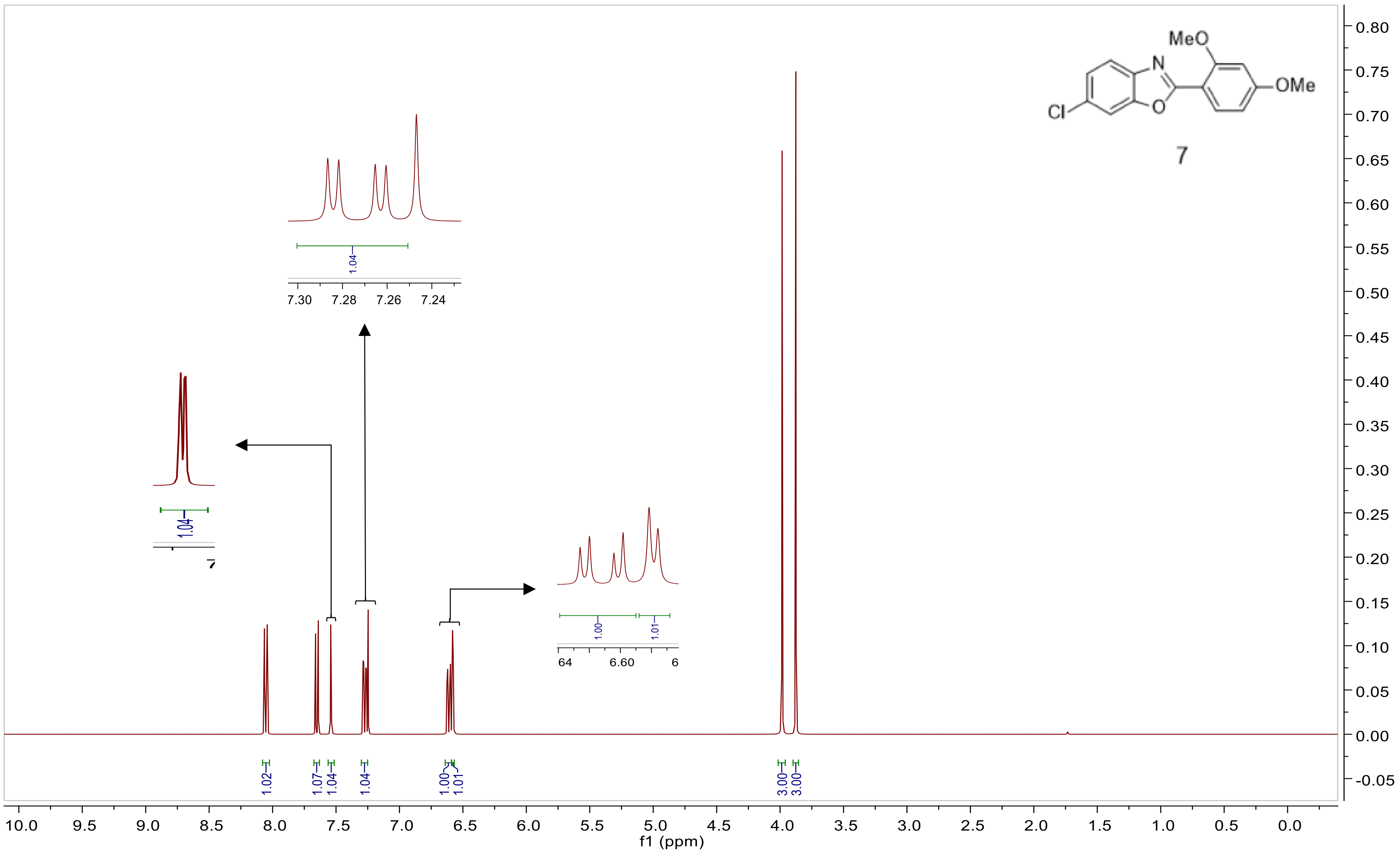

S13. ^{1}H NMR spectrum of analog **7**

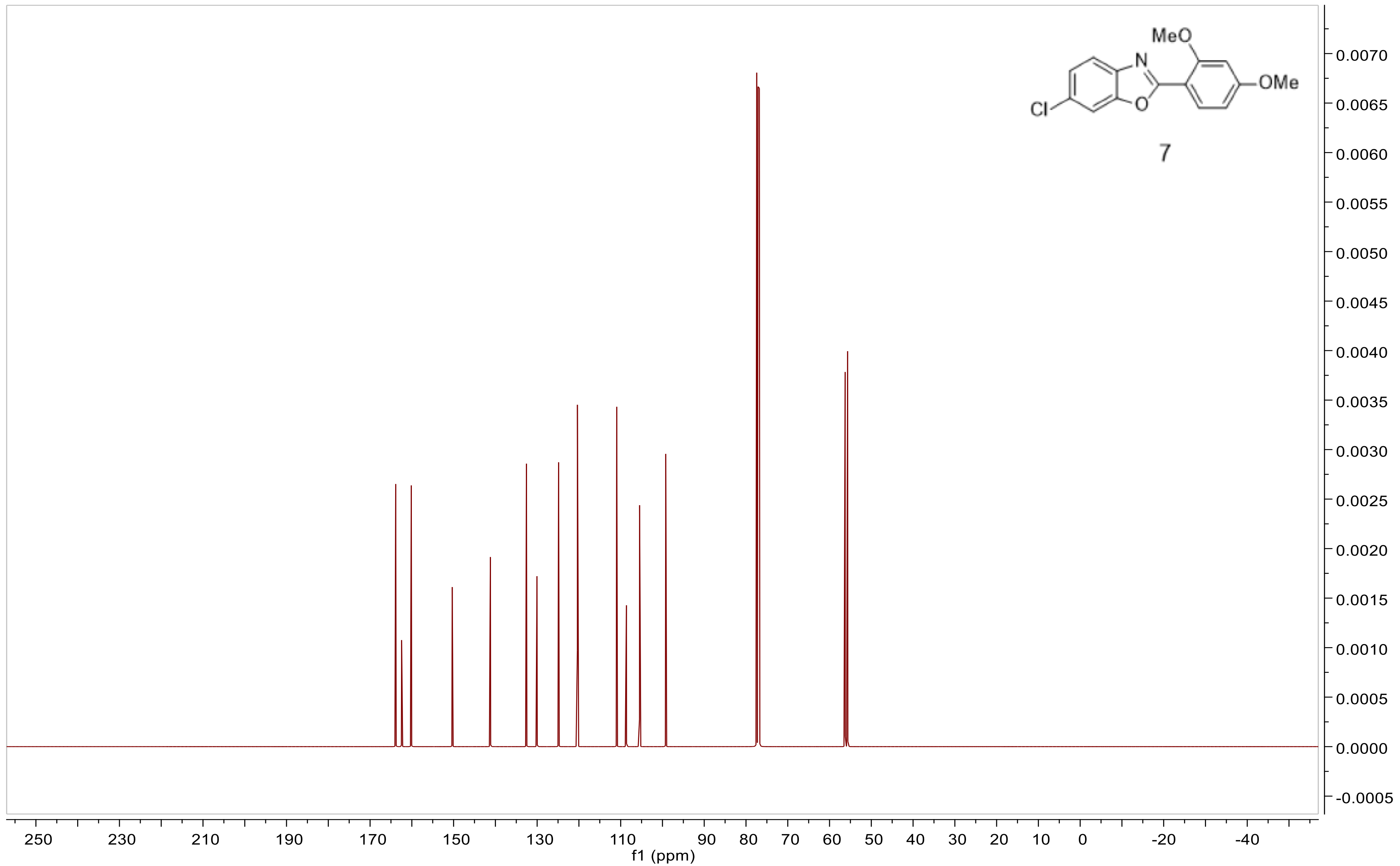

S14. ^{13}C NMR spectrum of analog **7**

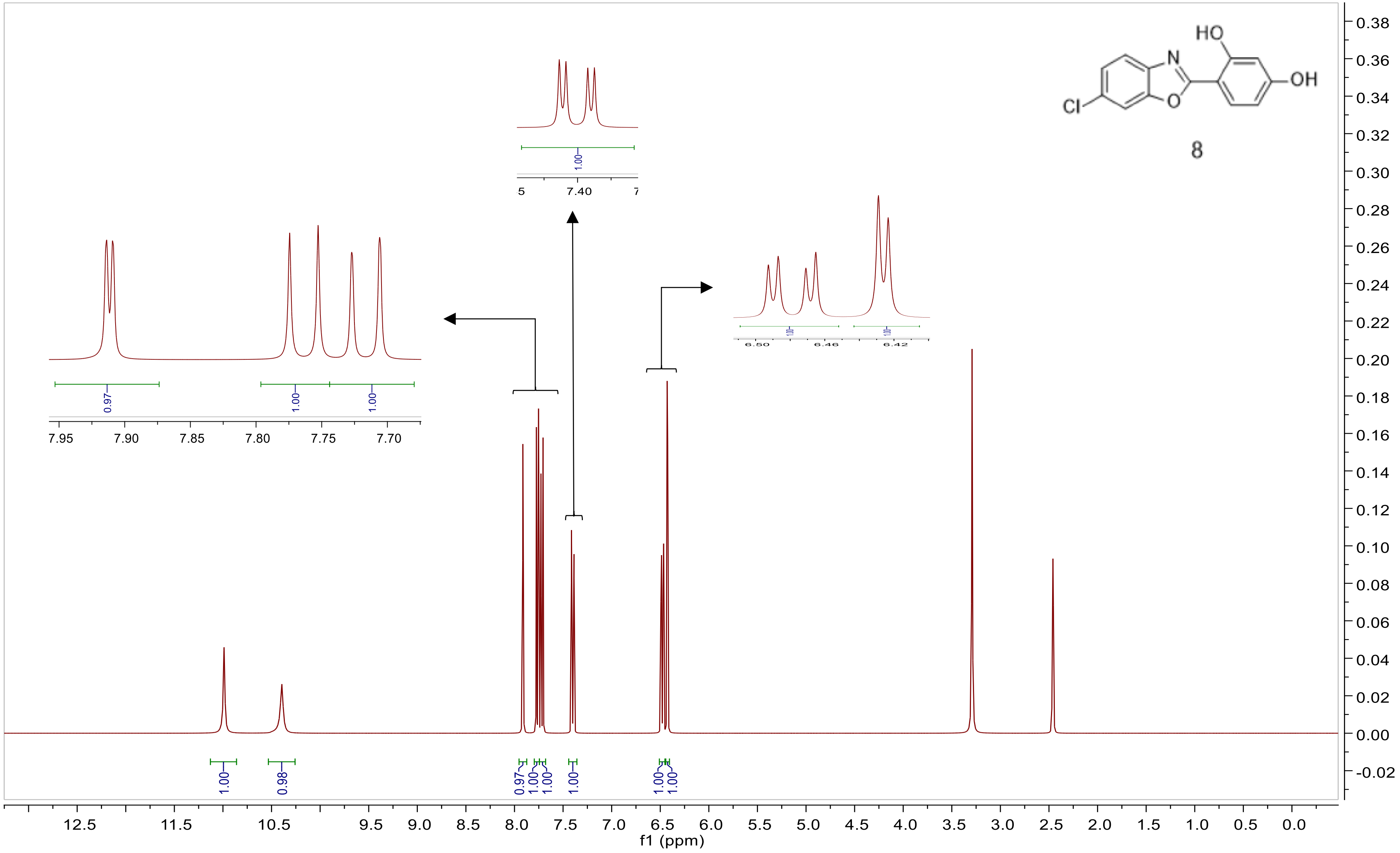

S15. ^{1}H NMR spectrum of analog **8**

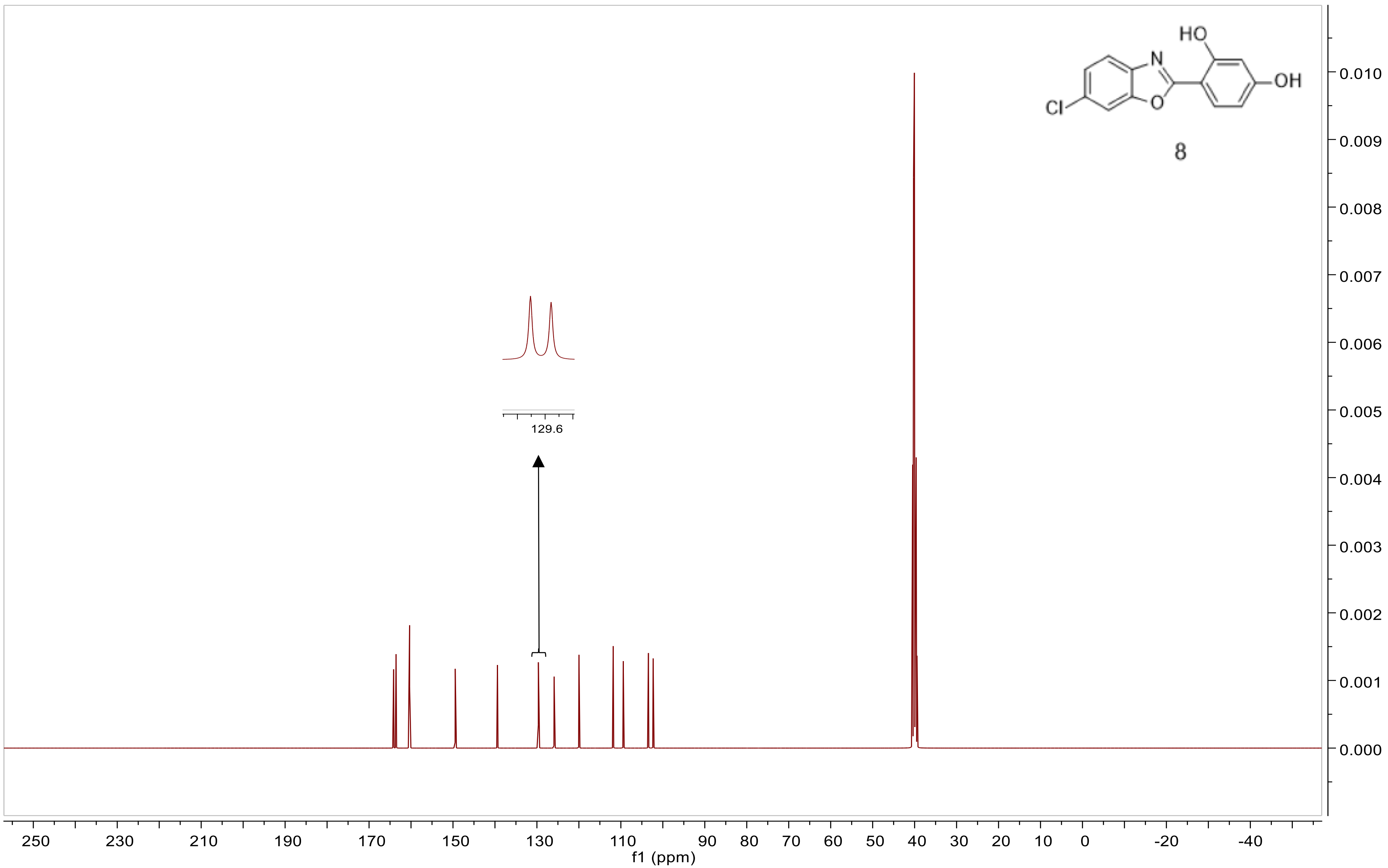

S16. ^{13}C NMR spectrum of analog **8**

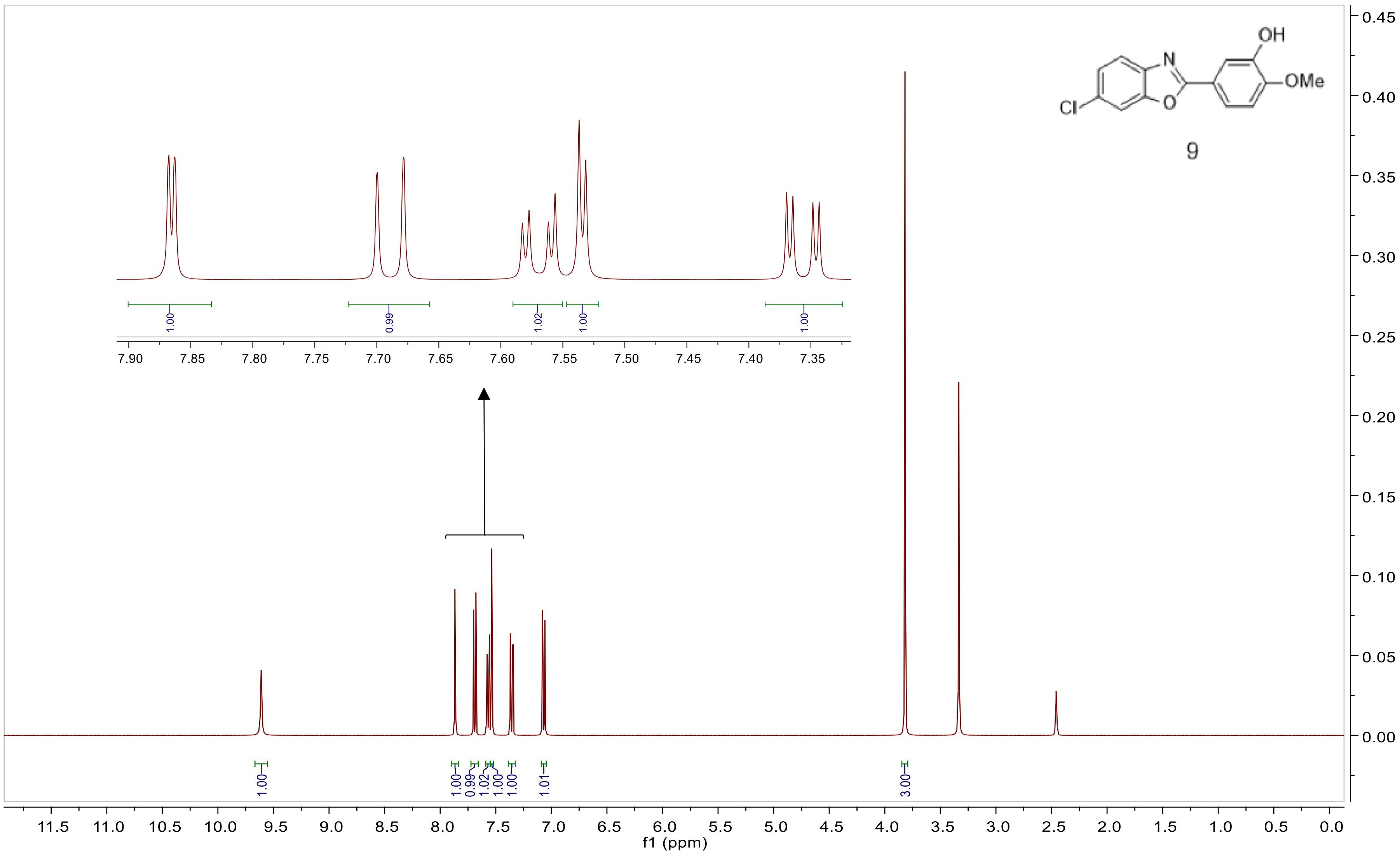

S17. ^{1}H NMR spectrum of analog **9**

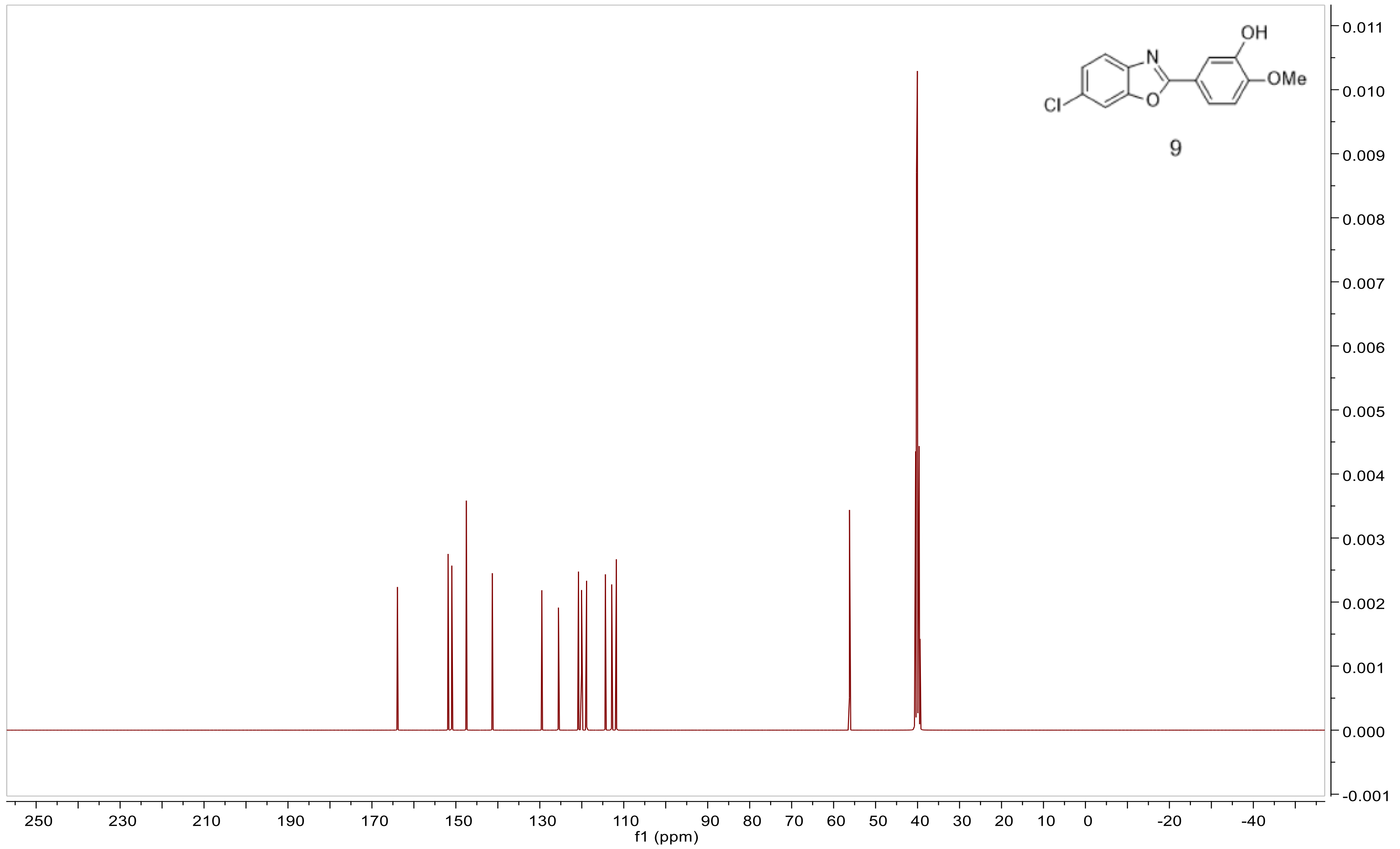

S18. ^{13}C NMR spectrum of analog **9**

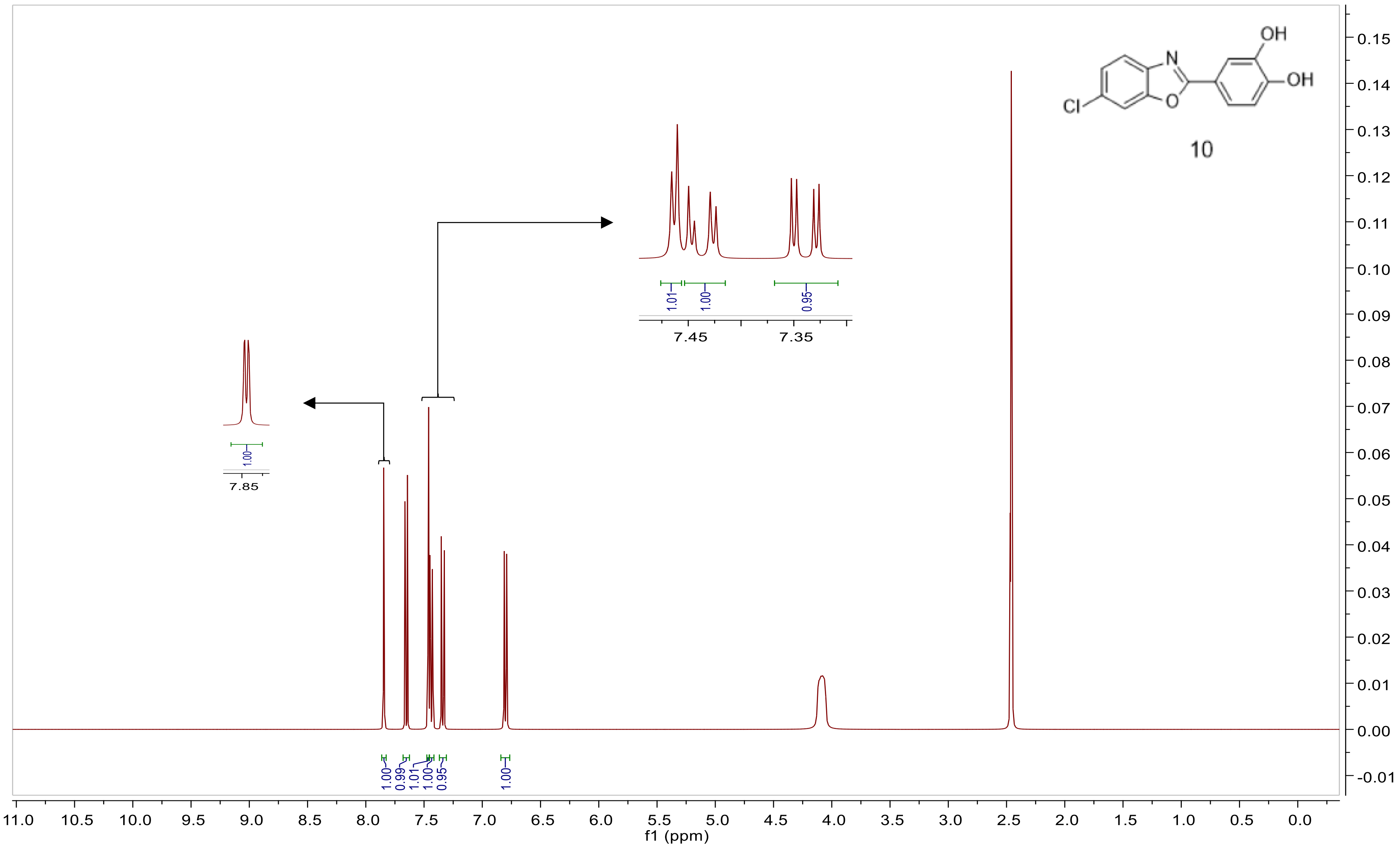

S19. ^{1}H NMR spectrum of analog **10**

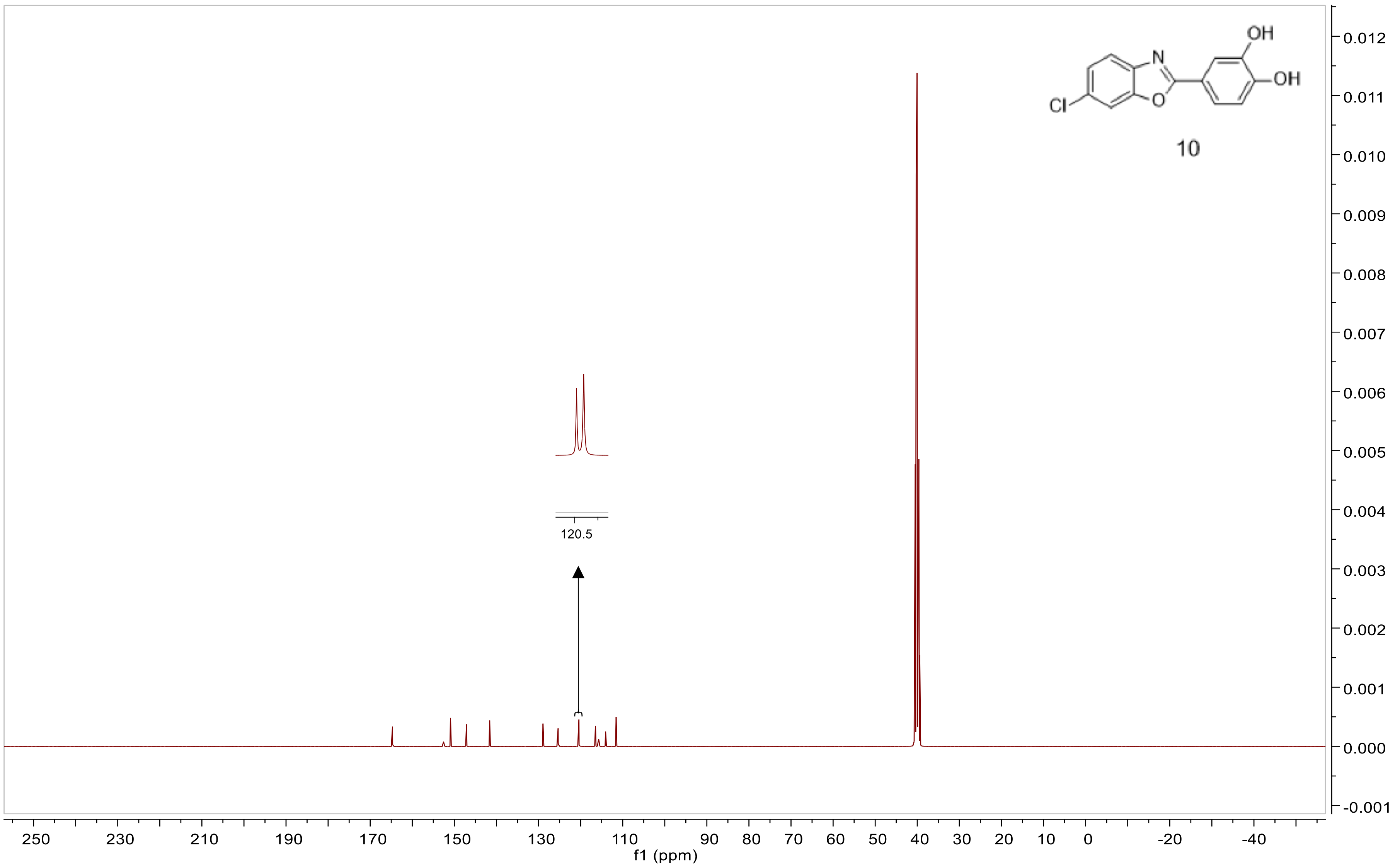

S20. ^{13}C NMR spectrum of analog **10**

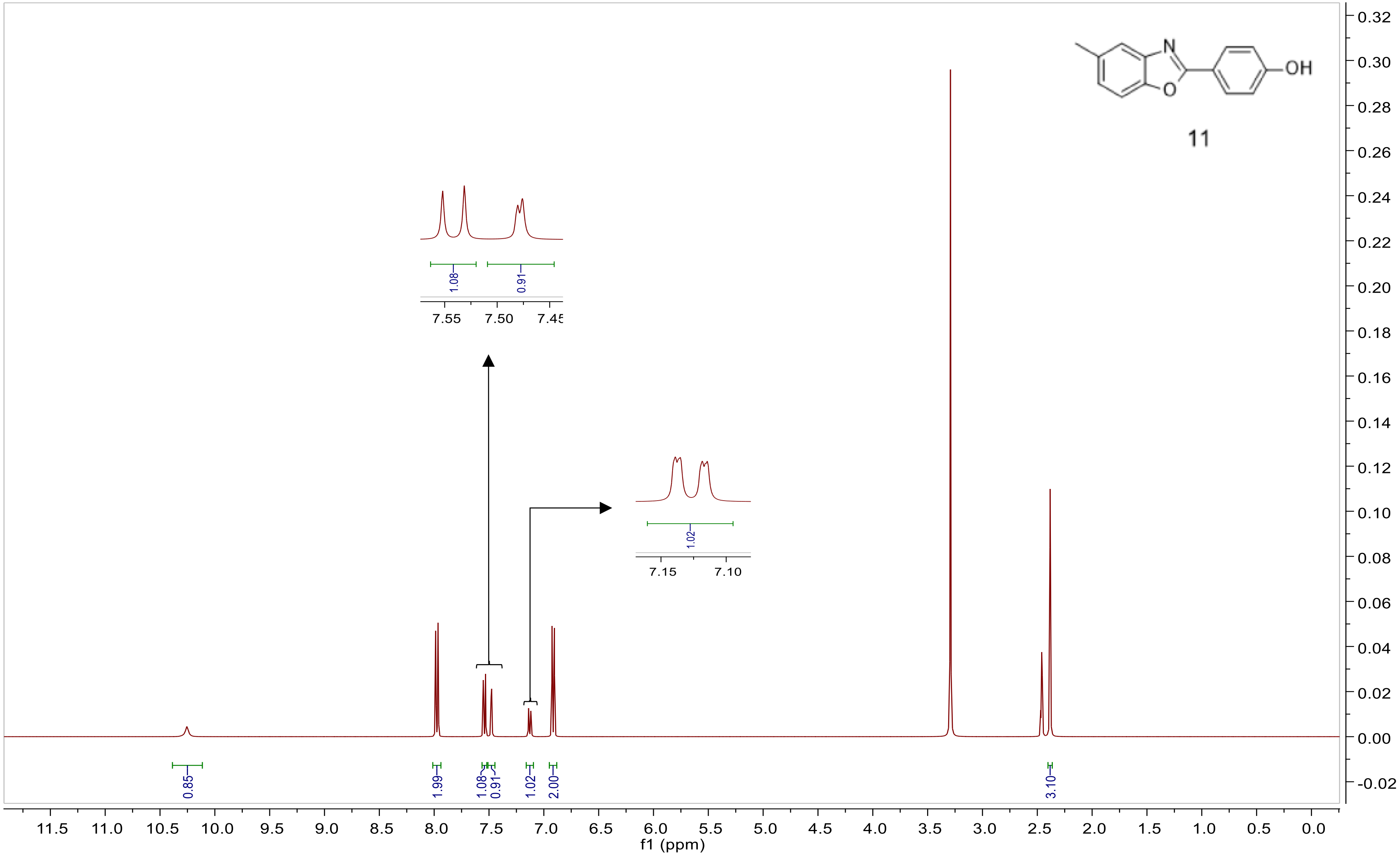

S21. ^{1}H NMR spectrum of analog **11**

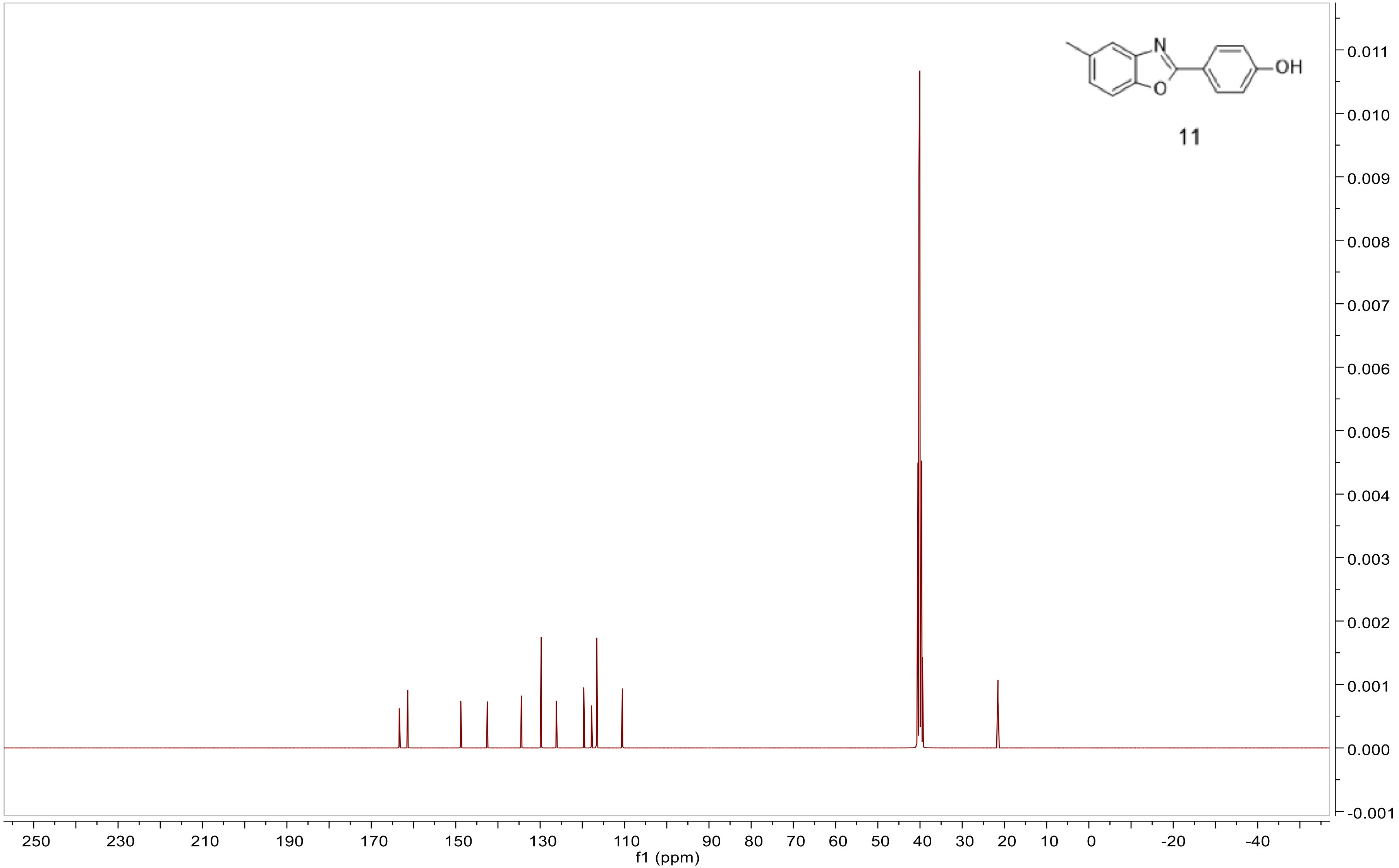

S22. ^{13}C NMR spectrum of analog **11**

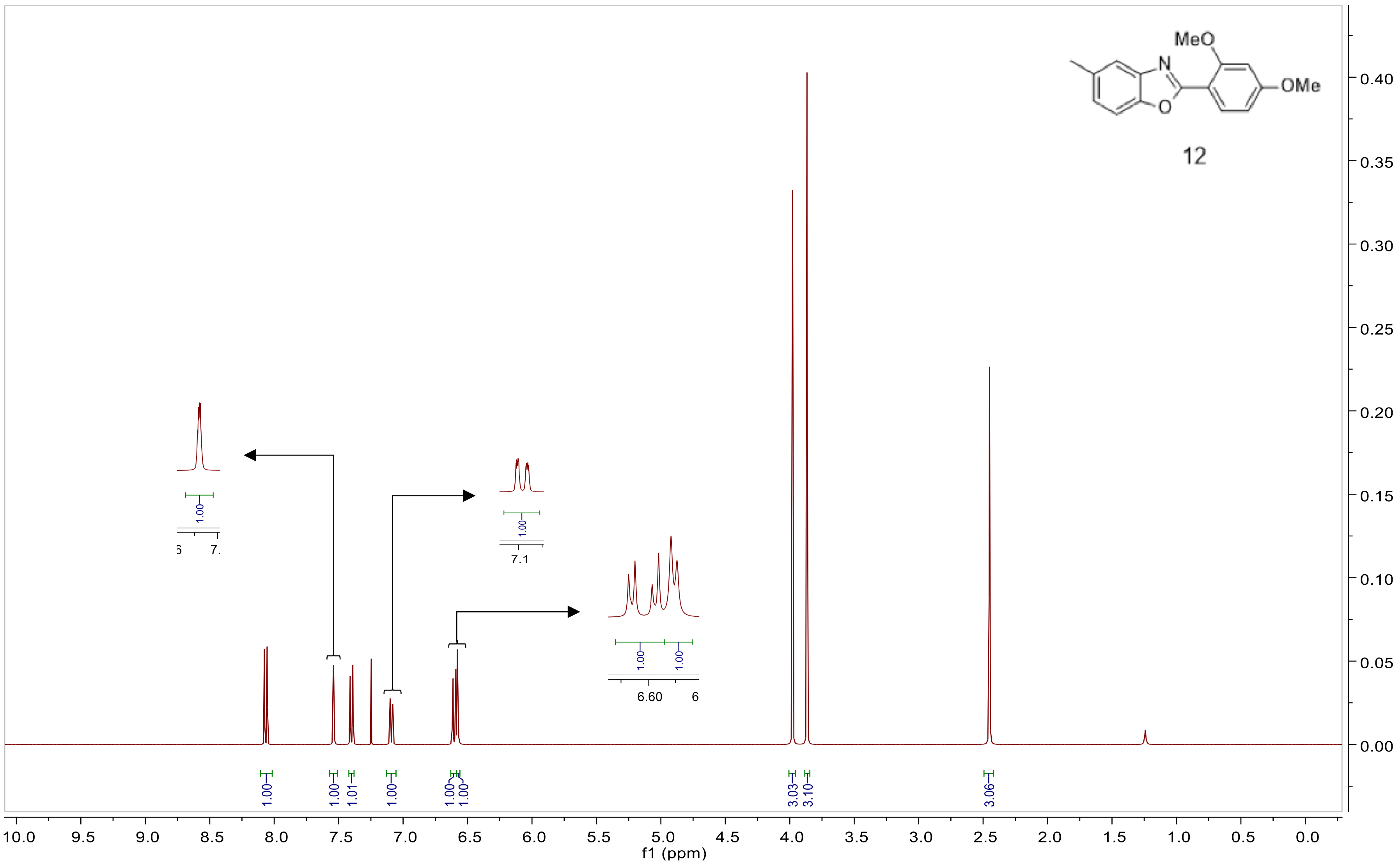

S23. ^{1}H NMR spectrum of analog **12**

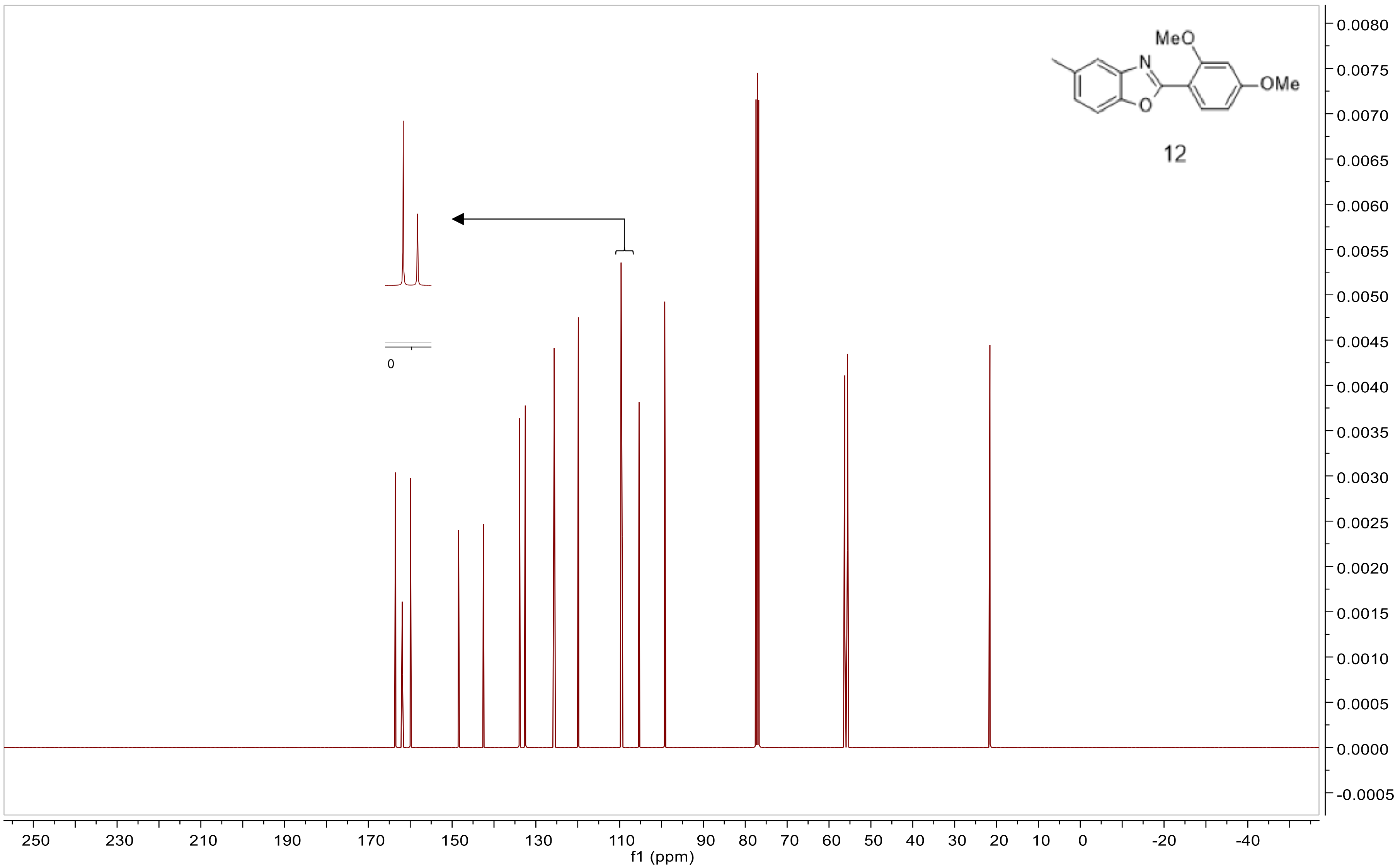

S24. ^{13}C NMR spectrum of analog **12**

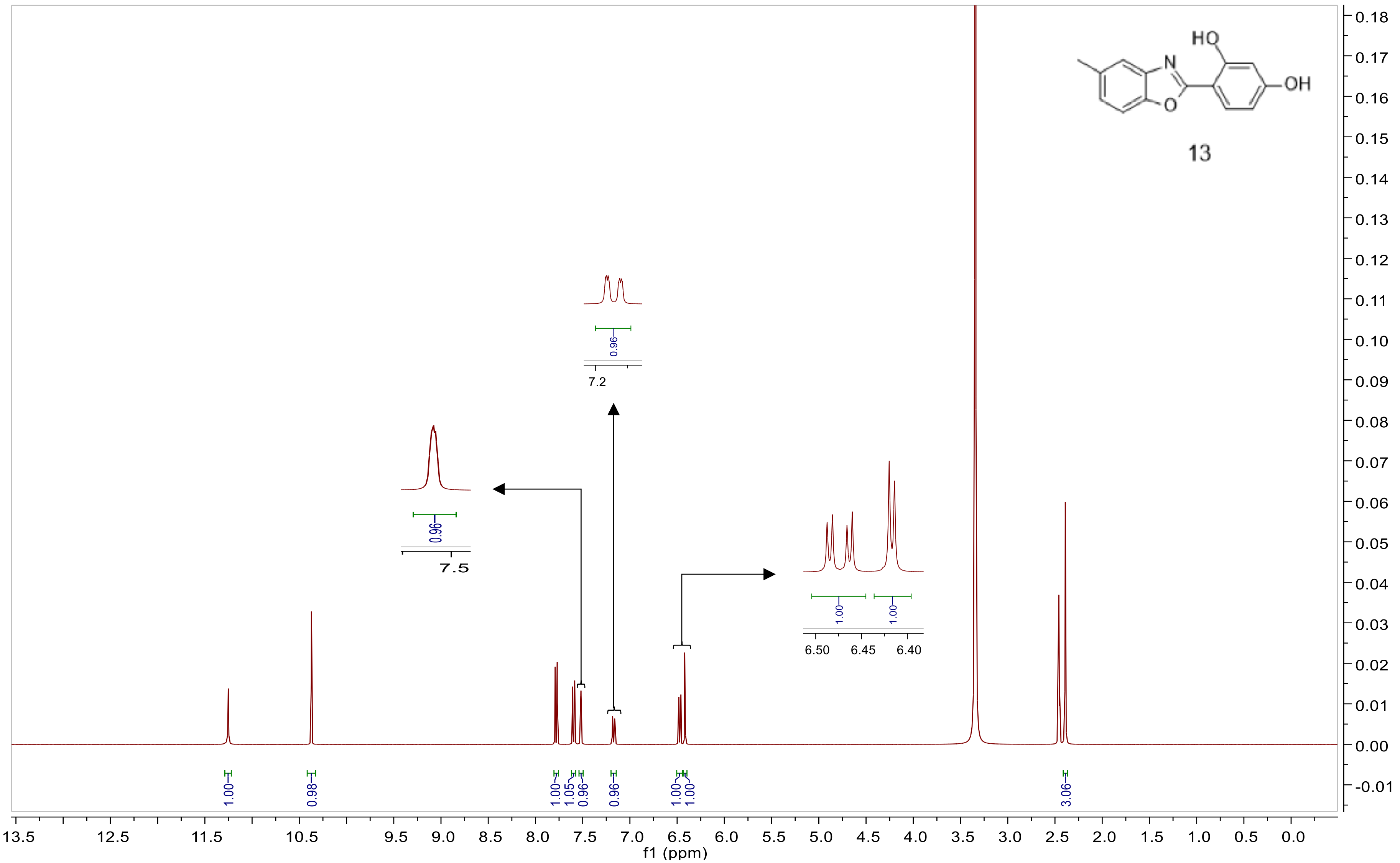

S25. ^{1}H NMR spectrum of analog **13**

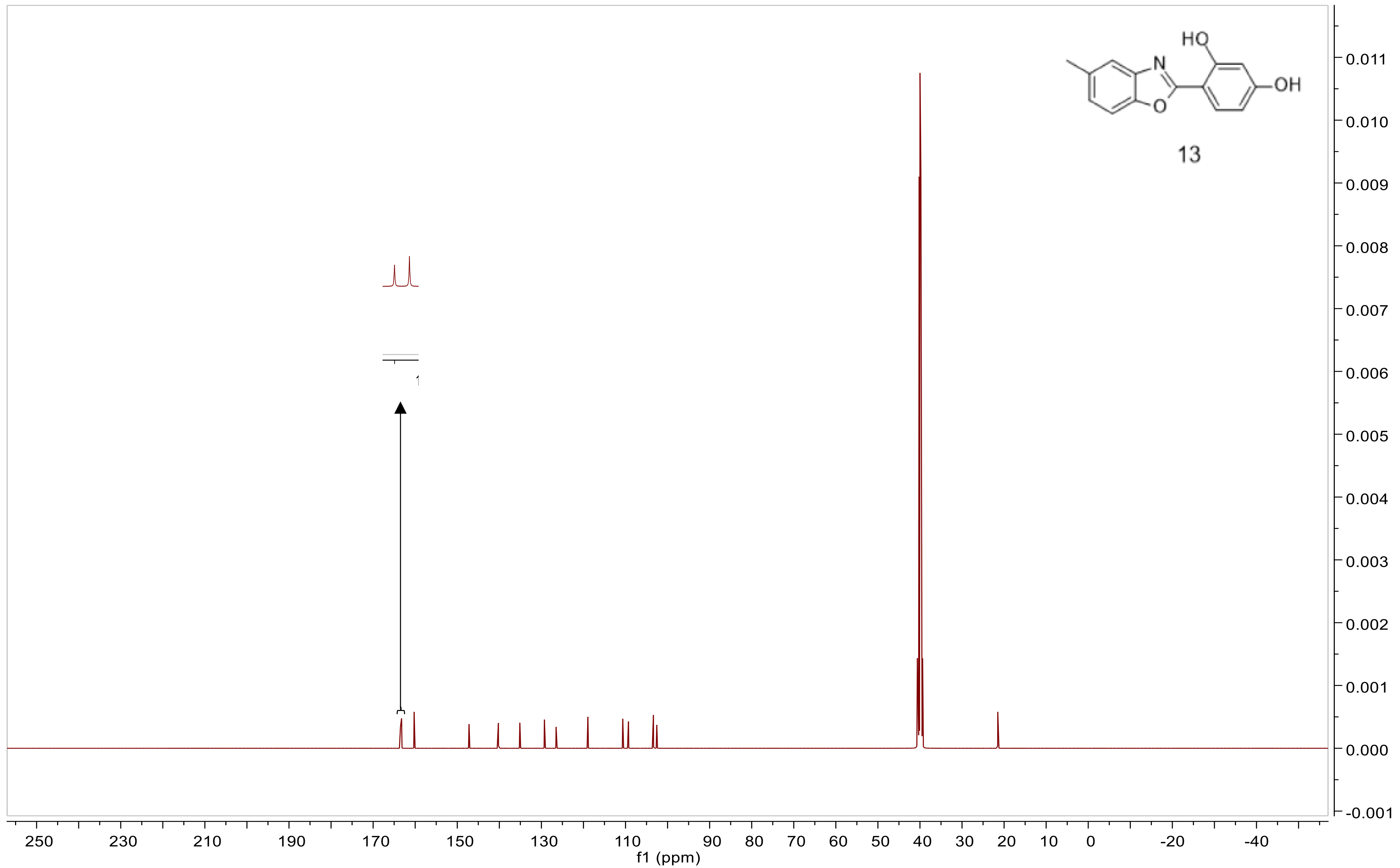

S26. ^{13}C NMR spectrum of analog **13**

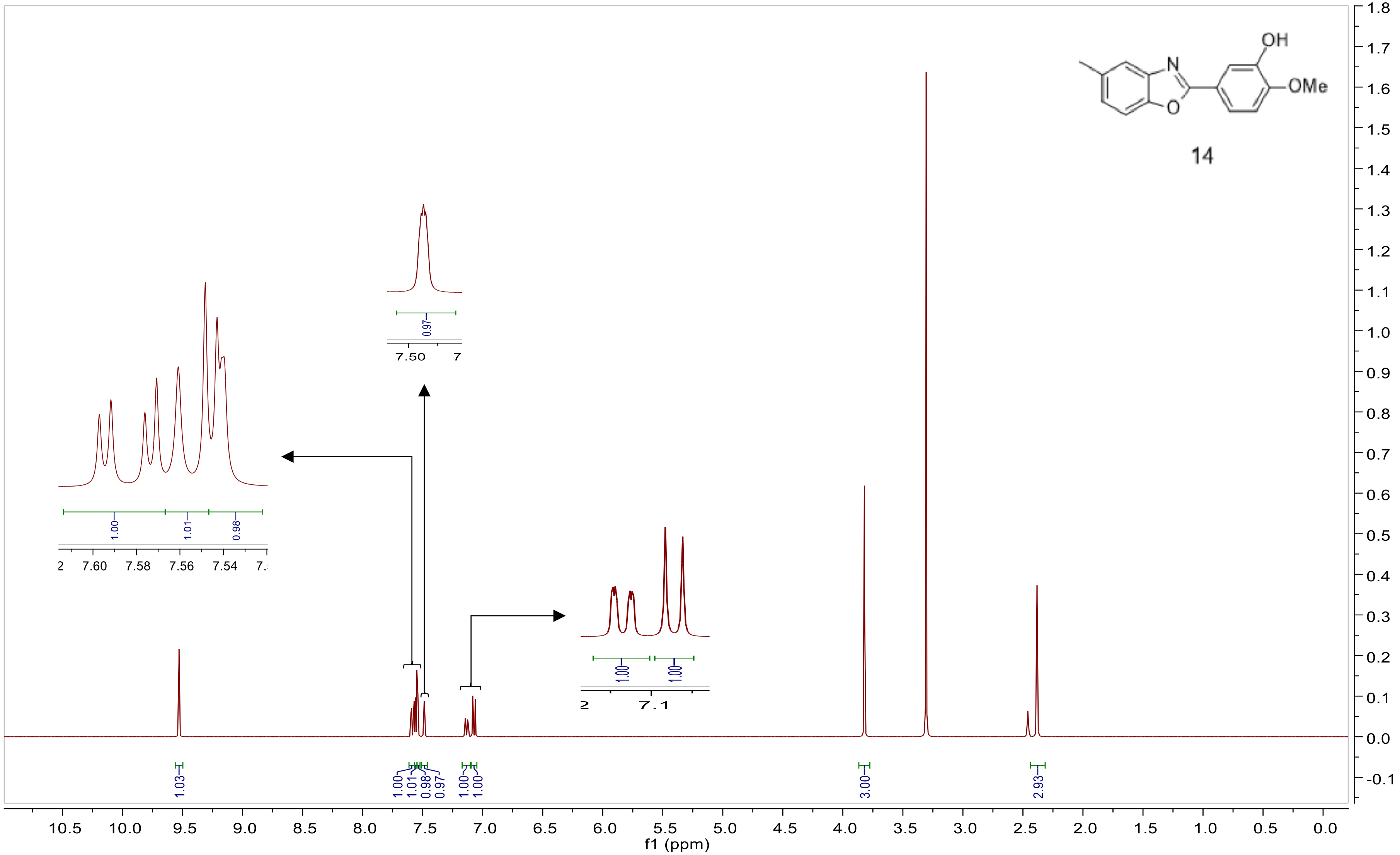

S27. ^{1}H NMR spectrum of analog **14**

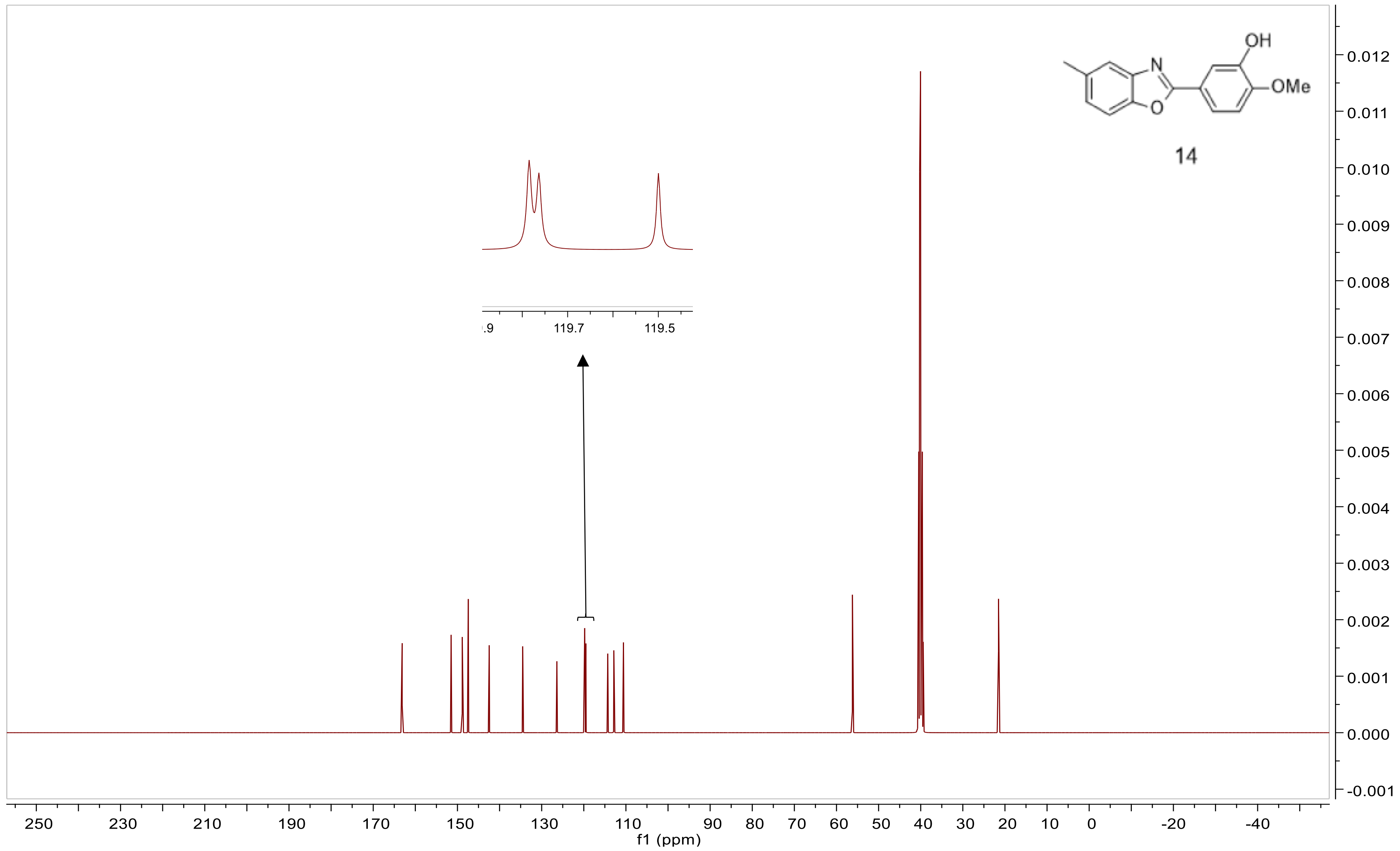

S28. ^{13}C NMR spectrum of analog **14**

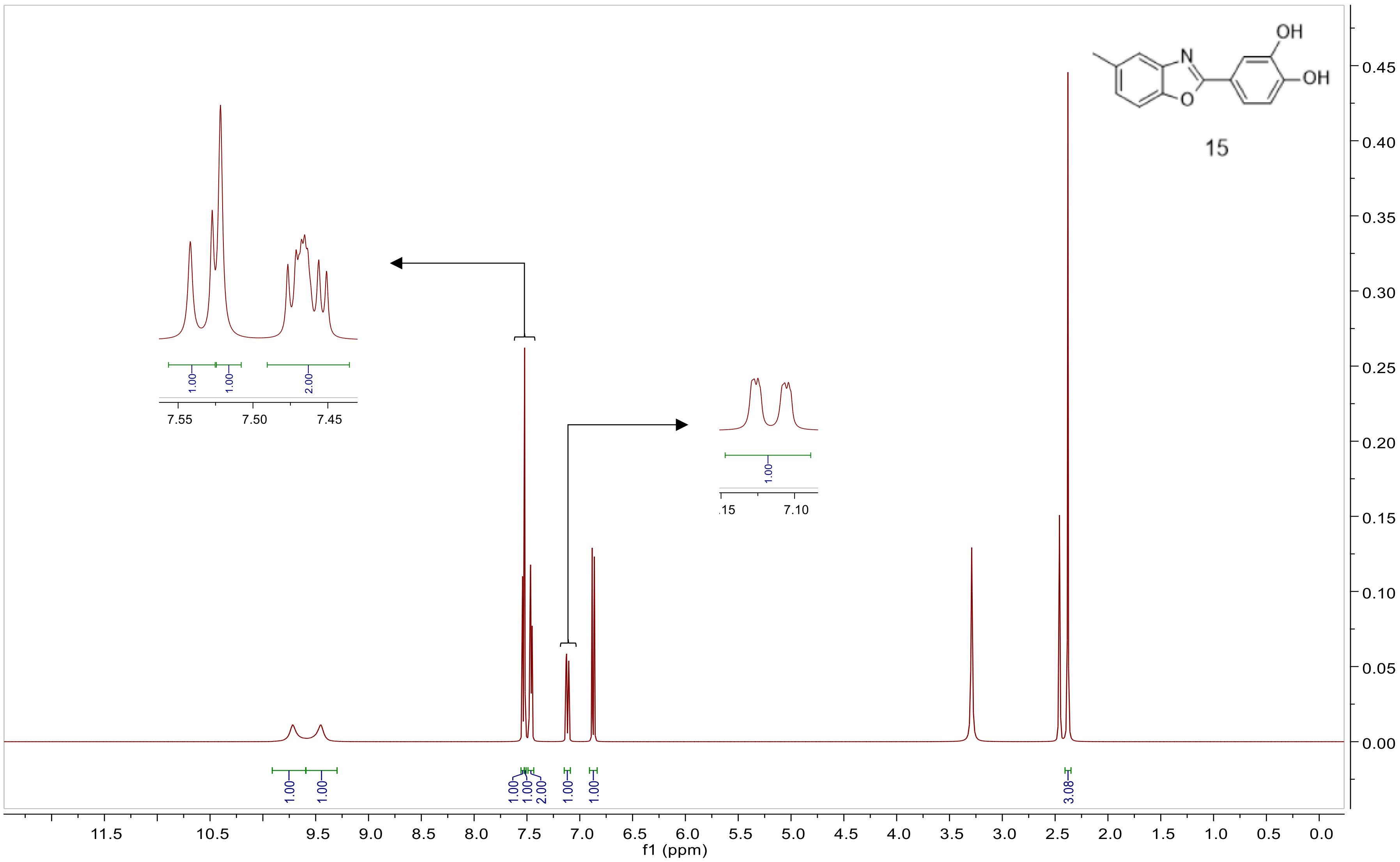

S29. ¹H NMR spectrum of analog **15**

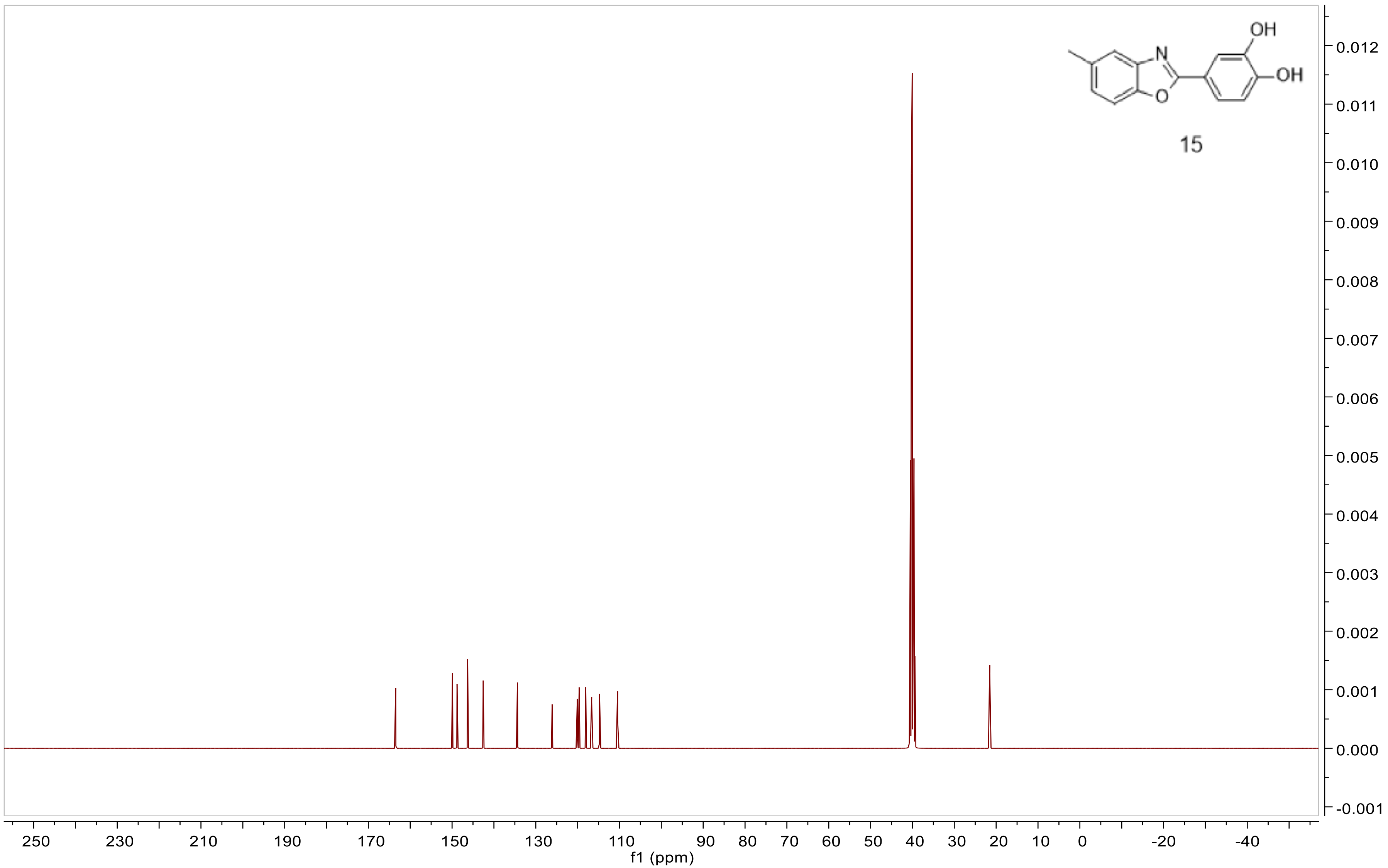

S30. ^{13}C NMR spectrum of analog **15**
